# Unification of *de novo* and acquired ibrutinib resistance in mantle cell lymphoma

**DOI:** 10.1038/ncomms14920

**Published:** 2017-04-18

**Authors:** Xiaohong Zhao, Tint Lwin, Ariosto Silva, Bijal Shah, Jiangchuan Tao, Bin Fang, Liang Zhang, Kai Fu, Chengfeng Bi, Jiannong Li, Huijuan Jiang, Mark B. Meads, Timothy Jacobson, Maria Silva, Allison Distler, Lancia Darville, Ling Zhang, Ying Han, Dmitri Rebatchouk, Maurizio Di Liberto, Lynn C. Moscinski, John M. Koomen, William S. Dalton, Kenneth H. Shain, Michael Wang, Eduardo Sotomayor, Jianguo Tao

**Affiliations:** 1 Departments of Chemical Biology and Molecular Medicine Program, Moffitt Cancer Center, Tampa, Florida 33612, USA; 2 Department of Cancer Imaging and Metabolism, Moffitt Cancer Center, Tampa, Florida 33612, USA; 3 Department of Malignant Hematology, Moffitt Cancer Center, Tampa, Florida 33612, USA; 4 Department of Laboratory Medicine and Hematopathology, Moffitt Cancer Center, Tampa, Florida 33612, USA; 5 Proteomics Core Facility, Moffitt Cancer Center, Tampa, Florida 33612, USA; 6 Department of Lymphoma/Myeloma, University of Texas MD Anderson Cancer Center, Houston, Texas 77030, USA; 7 Department of Pathology, University of Nebraska Medical Center, Omaha, Nebraska 68198, USA; 8 Biostatistics Core Facility, Moffitt Cancer Center, Tampa, Florida 33612, USA; 9 Department of Hematology, Tianjin Medical University General Hospital, Tianjian 300052, China; 10 Department of Biotherapy, Tianjin Medical University Cancer Institute and Hospital, National Clinical Research Center of Cancer, Key Laboratory, Tianjing 300040, China; 11 nPharmakon LLC, Piscataway, New Jersey 08854, USA; 12 Department of Pathology and Laboratory Medicine, Weill Cornell Medicine, New York, New York 10065, USA; 13 Department of Hematology & Oncology, George Washington University, Washington DC 20052, USA

## Abstract

The novel Bruton's tyrosine kinase inhibitor ibrutinib has demonstrated high response rates in B-cell lymphomas; however, a growing number of ibrutinib-treated patients relapse with resistance and fulminant progression. Using chemical proteomics and an organotypic cell-based drug screening assay, we determine the functional role of the tumour microenvironment (TME) in ibrutinib activity and acquired ibrutinib resistance. We demonstrate that MCL cells develop ibrutinib resistance through evolutionary processes driven by dynamic feedback between MCL cells and TME, leading to kinome adaptive reprogramming, bypassing the effect of ibrutinib and reciprocal activation of PI3K-AKT-mTOR and integrin-β1 signalling. Combinatorial disruption of B-cell receptor signalling and PI3K-AKT-mTOR axis leads to release of MCL cells from TME, reversal of drug resistance and enhanced anti-MCL activity in MCL patient samples and patient-derived xenograft models. This study unifies TME-mediated *de novo* and acquired drug resistance mechanisms and provides a novel combination therapeutic strategy against MCL and other B-cell malignancies.

## Abstract (web summary)

Ibrutinib has demonstrated high response rates in B-cell lymphomas but a lot of ibrutinib-treated patients relapse with resistance. This study unified TME-mediated *de novo* and acquired drug resistance through B-cell receptor signalling and PI3K-AKT-mTOR axis and provides a combination therapeutic strategy against B-cell malignancies.

Mantle cell lymphoma (MCL) is an aggressive B-cell lymphoma that accounts for ∼6–8% of all B-cell lymphomas. Prognosis remains poor in MCL patients due to the emergence of drug resistance and lymphoma progression^1^. MCL depends on the strong interactions between lymphoma cells and their tumour microenvironment (TME)^2^,^3^. Integrin β1-containing receptors (α4β1 and α5β1) are highly expressed in MCL cells and are major mediators of cell adhesion to stroma, provide protection against drug-induced apoptosis, and confer environment-mediated drug resistance (EMDR)^3^. Recently, the B-cell receptor (BCR) has emerged as a pivotal pathway in many B-cell lymphomas^4^,^5^. Upon activation of BCR, CD79 is phosphorylated, triggering a signalling cascade that involves activation of kinases, GTPases and transcription factors via a number of downstream pathways such as Bruton's tyrosine kinase (BTK), PI3K-AKT, ERK and NF-κB, promoting lymphomagenesis^6^. Inhibitors of BCR signalling have emerged as promising therapeutic agents for various B-cell lymphomas^7^,^8^,^9^. Ibrutinib is a novel BTK inhibitor that has shown an unprecedented overall response rate and progression-free survival in relapsed/refractory MCL patients and in patients with other B-cell disorders^10^,^11^. Clinically, ibrutinib rapidly induces lymphocytosis and lymph node shrinkage, a phenomenon common to BCR inhibitors, likely attributed to attenuation of BCR-dependent lymphoma–TME interactions^12^,^13^,^14^,^15^. Unfortunately, despite the dramatic responses to ibrutinib, resistance inevitably develops. Approximately 43% of MCL patients have shown partial or complete lack of response to ibrutinib and experienced disease progression within 12 months of treatment. Alarmingly, once patients relapse after ibrutinib treatment, the 1-year survival rate is only 22% (refs 16, 17). Similar outcomes have been reported in patients with chronic lymphocytic leukaemia after ibrutinib discontinuation because of disease progression and drug resistance^18^.

Drug resistance is generally considered to evolve by intrinsic or acquired genetic alterations and is heavily influenced by the extrinsic TME^3^. TME-mediated resistance is a form of *de novo* drug resistance that protects tumour cells from the effects of diverse therapies. Acquired resistance to kinase inhibitors is common and complex, involving mutations, reprogramming and reactivation of key intracellular signal networks^19^,^20^. However, the manner in which the TME contributes to the development of acquired ibrutinib resistance (IR) is largely unknown. To capture the complexity of IR, we applied activity-based protein profiling (ABPP) to examine the kinome response profiles in MCL modulated by stroma and/or chronic ibrutinib treatment. We interrogated TME-mediated and acquired drug resistance to determine the mechanistic link between TME and acquired IR. Combining kinomics, longitudinal drug screening with *ex vivo*, *in vivo* TME, and patient-derived xenograft (PDX) models, we identified a major kinase network involving PI3K-AKT-mTOR/integrin β1-integrin-linked kinase (ILK) as a central hub for TME–lymphoma interactions mediating IR. We found that combined disruption of BCR signalling and central pathways resulting from kinome reprogramming is critical for overcoming IR in MCL.

## Results

### BCR signal in TME–lymphoma interactions and drug resistance

**Figure 1:**
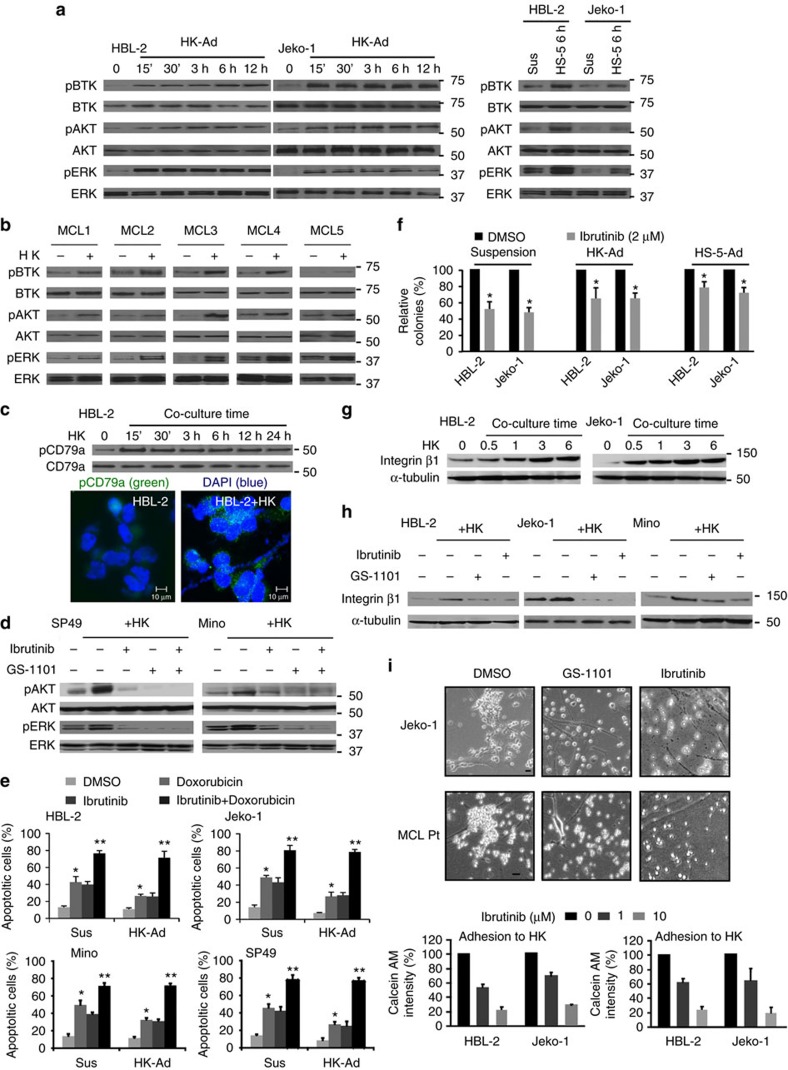
B-cell receptor (BCR) signalling is a central ‘outside-in' and ‘inside-out' signalling hub for MCL cell survival and growth. (**a**) Co-culture with lymph node stromal cells (HK-Ad) and bone marrow stromal cells (HS-5) induced a time-dependent BCR signalling activation detected by western blot for BCR downstream molecules (BTK, AKT, ERK) phosphorylation in MCL cell lines (HBL-2, Jeko-1). (**b**) Co-culture with lymph node stromal cells induced BCR signalling activation detected by western blot for BCR downstream molecules (BTK, AKT, ERK) phosphorylation in primary MCL samples (MCL1–5). (**c**) Co-culture with lymph node stromal cells induced BCR (CD79a) phosphorylation detected by western blot and immunofluorescence staining in HBL-2 cells in co-culture with HK cells. Scale bar, 10 μM. (**d**) Inhibition of BCR signalling using BTK and PI3Kδ inhibitors (50 nM ibrutinib and 100 nM GS-1101) attenuated stroma-induced AKT and ERK activation in SP49 and Mino cells. (**e**) Ibrutinib induced cell apoptosis, overcame stroma-mediated drug resistance and enhanced doxorubicin (0.5 μM)-induced apoptosis in Jeko-1, HBL-2, Mino and SP49 cells (Sus, cells in suspension, HK-Ad, cells in co-culture with HK cells). *P<0.05 and **P>0.05 (Student's *t*-test). (**f**) Ibrutinib inhibited clonogenic growth with and without stroma co-culture. (**g**) Co-culture of stromal HK cells with MCL cells upregulated β1 expression in HBL-2 and Jeko-1 cells. (**h**) Inhibition of BCR signalling using BTK and PI3Kδ inhibitors (1 μM ibrutinib and 1 μM GS-1101) attenuated stroma-induced integrin β1 expression in HBL-2, Jeko-1 and Mino cells. (**i**) Inhibition of BCR signalling by ibrutinib attenuated adhesion of HBL-2, Jeko-1 and MCL patient cells to stroma cells (HK or HS-5) determined by microscopic examination and by the intensity of calcein-AM. Results in **a**–**i** are representative or means±s.d. from at least three biological replicates. See also Supplementary Figs 1, 8 for full gel scan of the WB.

We investigated the role of BCR signalling in stroma-mediated MCL cell survival and drug resistance and used a co-culture model to evaluate the impact of stromal cells on phosphorylation status of the BCR downstream proteins CD79a, BTK, ERK and AKT. As shown in Fig. 1a,b, co-culture of MCL cells with lymph node stromal cells (HK cells) or bone marrow stromal cells (HS-5) significantly increased pBTK, pERK and pAKT in MCL cell lines (HBL-2 and Jeko-1) and primary MCL cells. Consistent with BCR activation, stroma-induced phosphorylation of CD79a was observed (Fig. 1c). When CD79a was depleted by using shRNA, stroma-induced activation of BTK and AKT was abolished (Supplementary Fig. 1a), supporting that BCR is required for stroma-induced activation of BTK, ERK and AKT.

Next, we used selective BTK and PI3Kδ inhibitors (ibrutinib and GS-1101, respectively) to explore the functional role of BCR signalling in MCL survival and stroma-mediated drug resistance. Indeed, both ibrutinib and GS-1101 abolished intrinsic and stroma-induced activation of BTK, AKT and ERK (Fig. 1d, Supplementary Fig. 1b). Ibrutinib induced cell apoptosis, sensitized responses to the cytotoxic agents and suppressed MCL survival and clonogenic growth with and without stroma co-culture. In addition, BCR signalling pathway inhibition overcame stroma-mediated drug resistance (Fig. 1e,f, Supplementary Fig. 1c,d). These data indicate that TME/stroma trigger a sustained ‘outside-in' BTK, ERK and AKT activation via a stroma-induced BCR signal and with enhanced MCL survival/drug resistance.

BCR activation in the context of stroma may be secondary to a number of factors. To address how BCR is activated after co-culturing with stroma cells, we evaluated whether stroma-secreted proteins regulate BCR activation in MCL. LC-MS/MS-based label-free proteomics experiments were performed to quantify the secreted proteins (secretome) in HK cells. We detected many cytokines (IL3, IL8, IL10, CCL3, CCL4), growth factors (TGF-β, VEGF, HDGF, IGF2, PDGF) and extracellular matrix proteins (fibronectin, collagen, MMP1/3, laminin) in the supernatant of stromal HK cells. We identified 187 proteins in the HK cell culture supernatant. By gene enrichment analysis, the most enriched and shared proteins in the HK supernatant were extracellular matrix proteins and adhesion/chemokine molecules (Supplementary Fig. 1e).

Given that fibronectin binds to integrin β1 (β1) at the cell surface and relays protective environmental signals to cells, we examined changes in BCR signalling after MCL cells were adhered to fibronectin-coated plates. Indeed, MCL cell (Jeko-1, HBL-2) adhesion to fibronectin triggered a rapid induction of BCR signalling activation manifested by increased phosphorylation of CD79a, BTK, AKT and ERK (Supplementary Fig. 1f). Because our previous work revealed that lymphoma stroma-derived B-cell activating factor (BAFF) confers stroma-mediated protection of MCL cells from therapy-induced apoptosis^21^, we examined whether BAFF also activates the BCR pathway. Addition of BAFF to MCL cell lines and to primary B lymphocytes induced CD79a, BTK, ERK and AKT phosphorylation. More importantly, neutralization of BAFF with soluble TACI attenuated stroma-induced BCR activation (Supplementary Fig. 1g), indicating that BAFF-like fibronectin may contribute to stroma-induced BCR activation. Collectively, these data indicate that BCR activation by stroma is multifactorial and are in line with previous studies demonstrating that BCR and its downstream proteins are activated by a variety of molecules, including BAFF, antigen receptors and integrins^22^,^23^,^24^.

We also explored the contribution of BCR signalling to β1 expression and MCL adhesion by co-culturing with HK or HS-5 stroma cells. Co-culture with HK or HS-5 cells and treatment with BAFF upregulated β1expression in MCL cells (Fig. 1g, Supplementary Fig. 1h). As shown in Fig. 1h,i, targeting BCR signalling with ibrutinib or GS-1101 blocked stroma-induced β1 expression and attenuated MCL adhesion to stroma in both MCL cell lines and primary MCL cells. We also found that inhibition of AKT significantly decreased β1 expression and MCL cell adhesion to stroma, otherwise constitutively AKT activation through myristoylated AKT plasmid (myr-Akt) transfection increased expression of β1 and cell adhesion in these MCL cells (Supplementary Fig. 1i,j). Thus, the PI3K-AKT signalling pathway is a central ‘outside-in' and ‘inside-out' signalling hub for stroma–lymphoma interactions and stroma-mediated drug resistance.

### Adaptive kinome reprogramming in acquired IR MCL

**Figure 2:**
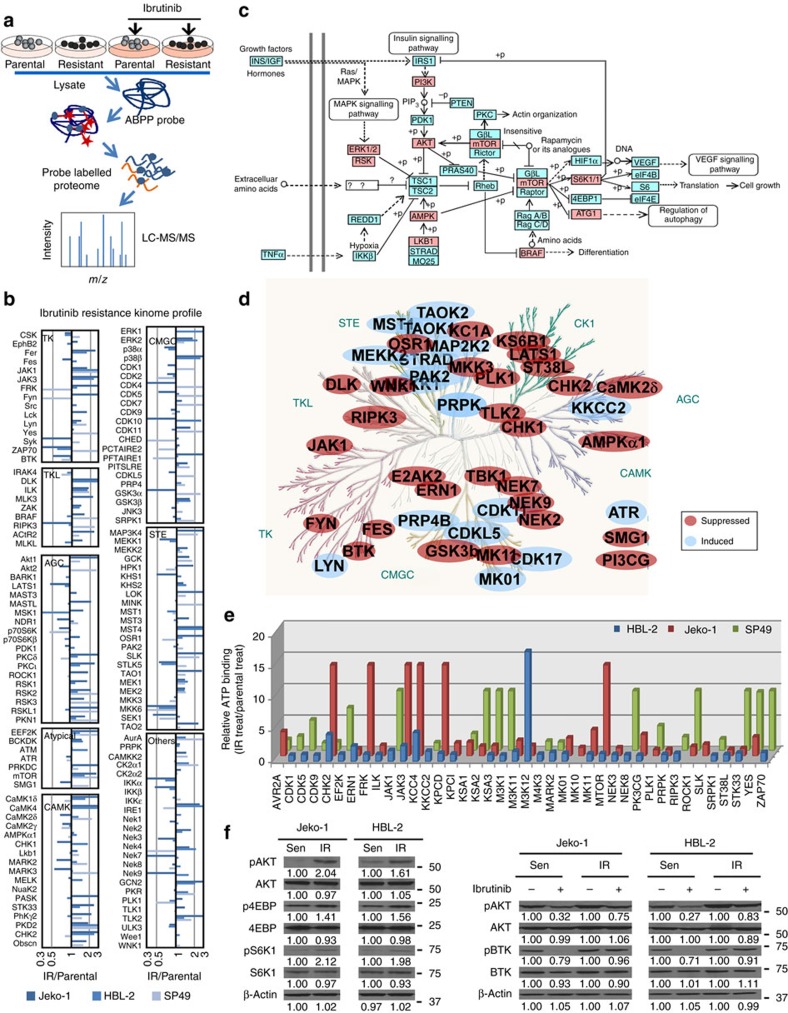
Chronic ibrutinib treatment leads to kinome reprogramming and PI3K-AKT-mTOR pathway activation and ibrutinib resistance in MCL cells. (**a**) Schematic workflow of activity-based protein profiling (ABPP) to identify global kinome changes in MCL cells. Briefly, cell lysis were sonicated, desalted and followed by incubation with 10 μM desthiobiotin-ATP probes. The labelled proteins were reduced, alkylated and trypsin digested, and the labelled peptides were purified with high capacity streptavidin agarose resin, washed, eluted and subjected to LC-MS/MS for quantification. (**b**) Kinome profiling of IR MCL cells compared with parental cells (HBL-2, Jeko-1, SP49). Ratios ≥1 indicate increased kinase ATP probe binding with relative increased activity, and values <1 indicate decreased ATP probe binding with decreased activity relative to parental cells. Data averages of two biological replicates. (**c**) KEGG pathway analysis map showing activation of PI3K-AKT-mTOR as a central signalling hub of kinome reprogramming for IR development. Pink, kinases with increased activity detected by ABPP. (**d**) Common kinase ATP-binding changes after 6-h ibrutinib treatment in all three parental MCL cell lines (Jeko-1, HBL-2, SP49). Kinome trees reproduced courtesy of Cell Signaling Technology. (**e**) Sustained kinase ATP-binding activity in IR MCL lines relative to their parental cell lines after 6 h ibrutinib treatment. (**f**) Western blots showing increased AKT, 4EBP and S6K1 phosphorylation in Jeko-1-IR and HBL-2-IR cell lines, as well as sustained AKT activation in the presence of ibrutinib (6 μM, 12 h) treatment in IR cells compared with parental (Sen) cells. (**b**,**d**,**e**) are from two biological replicates with four technical replicates. Results in **f** are representatives of three independent experiments. The relative changes of proteins were measured by quantitative densitometry and indicated below each lane. See also Supplementary Figs 2, 8 for full gel scan of the WB.

To investigate the IR mechanisms, we generated three independent IR MCL cell lines by growing Jeko-1, HBL-2 and SP49 cells in increasing concentrations of ibrutinib over 6 months (Supplementary Fig. 2a). First, Sanger sequencing for the C481S and R665W mutations in BTK and PLCG2 was performed in these three pairs of MCL lines respectively. Intriguingly, no C481S and R665W mutations in BTK and PLCG2 reported in IR chronic lymphocytic leukaemia were detected in all lines^19^,^20^,^25^,^26^. Next, we determined the key signalling adaptations associated with the development of acquired IR by performing ABPP to identify global kinome in both parental and IR cells. For ABPP, we used a desthiobiotin-ATP probe for selective labelling of active sites in ATP-binding proteins, which was followed by identification and quantification using liquid chromatography-tandem mass spectrometry (Fig. 2a). By using three paired parental and IR MCL cell lines, this approach identified 7,382 peptides corresponding to 2,327 ATP binding proteins, including 547 peptides corresponded to 250 protein kinases (Supplementary Fig. 2b). By extrapolating kinase expression in gene profiling data from the published data (GSE34602, GSE52148, GSE52434, GSE68091) in MCL lines (Jeko-1, HBL-2 and SP49), we concluded that there are 424 kinases present in MCL cells. Thus, the 250 kinases identified by our proteomic analysis accounted for approximately 59% of the expressed kinome of MCL cells (Supplementary Data 1).

Next, by comparing changes in relative kinase activities between IR and parental MCL cells at a system level, ABPP revealed that the kinome of IR cells was markedly changed, with many kinases displaying enhanced ATP probe binding in at least two cell lines, indicating increased activity relative to sensitive cells (Fig. 2b). These changes included biologically significant protein kinases such as CDKs, ILK, DNA damage response proteins and JAK-STAT kinases. By plotting the frequency distribution of the kinase changes and dividing the data into four quantiles, we selected fold changes of 1.35 for HBL-2, 1.92 for Jeko-1 and 2.28 for SP49 cells as cut-off values, as these values defined the top quantile changes within each cell line, and a significant overlap among these top quantile kinases was observed (Supplementary Fig. 2c,d). Further mapping of the overlapped kinases by using KEGG pathway analysis identified PI3K-AKT-mTOR pathways as a central signalling hub in IR MCL cells (Fig. 2c).

We next compared kinome alterations in sensitive and IR cells following ibrutinib treatment and determined the kinases/pathways that were inhibited with ibrutinib treatment in sensitive cells but remained unaltered or elevated in IR cells despite ibrutinib treatment. As illustrated in Fig. 2d, parental cells were responsive to ibrutinib, showing reduction of BTK binding in all three cell lines. We identified a kinome signature with significant changes in ATP binding after 6-h ibrutinib treatment. The changes in protein kinase activity by ibrutinib treatment could be attributed to both direct and indirect ibrutinib targets. As expected, IR cells displayed a more blunted response to ibrutinib than parental cells. Many of the kinases demonstrated unchanged or even increased activity in response to ibrutinib, supporting the concept that BTK-independent kinases or BTK bypass pathways drive IR (Fig. 2e and Supplementary Fig. 2e). Our analyses identified 40 kinases that remained unchanged or even elevated after ibrutinib treatment, including multiple PI3K-AKT-mTOR-related kinases (Fig. 2e). Because the PI3K-AKT-mTOR pathway was most frequently found in this setting (Fig. 2c), we further assessed activation of PI3K-AKT-mTOR and its downstream targets in parental and IR MCL cells. Among these, we found significantly increased AKT, 4EBP and S6K1 phosphorylation levels in IR cell lines compared with their parental cells (Fig. 2f). Ibrutinib effectively decreased AKT and BTK phosphorylation in parental MCL cells. In contrast, high AKT phosphorylation in IR MCL cell lines was sustained in the presence of ibrutinib treatment, suggesting a mechanism of bypassing the effect of ibrutinib to promote activation of the PI3K-AKT-mTOR axis.

### PI3K-AKT-mTOR pathway activation in IR MCL cells

**Figure 3:**
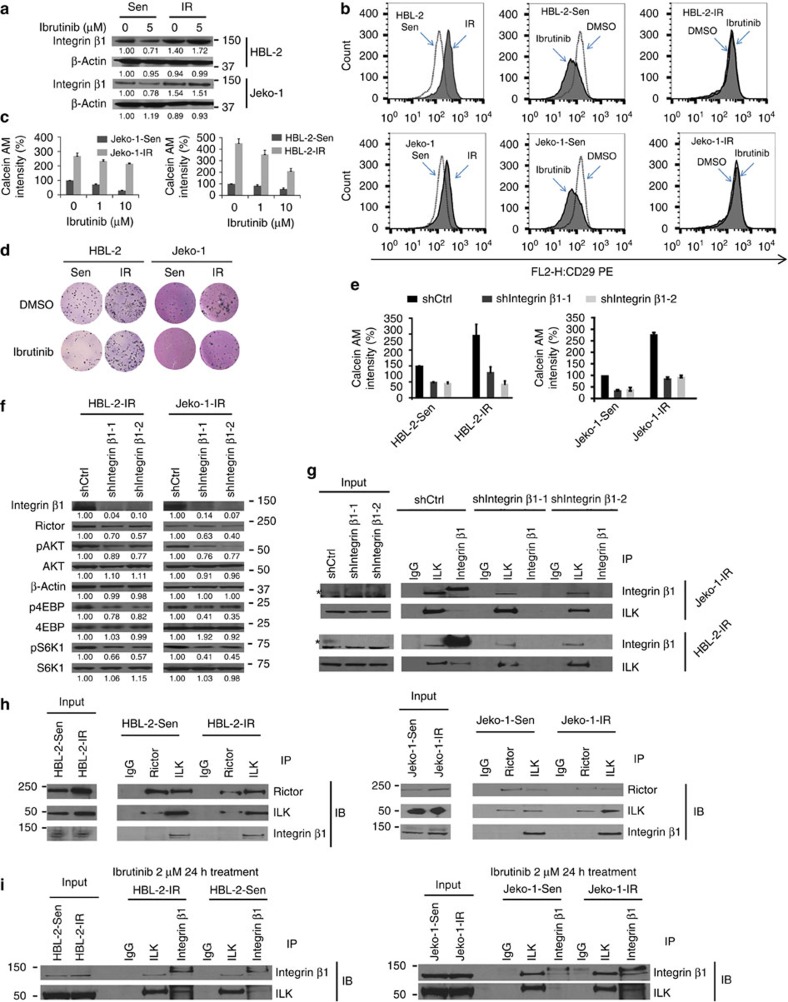
Interplay of PI3K-AKT-mTORC and integrin β1/integrin linked kinase pathways in IR MCL cells. (**a**,**b**) Western blots (**a**) and flow cytometry (**b**) showing increased and sustained integrin β (β1) expression in IR MCL cells compared to parental (Sen) cells in the absence and presence of ibrutinib treatment (6 h). (**c**) Cell adhesion assay showing increased and sustained cell adhesion of IR MCL cells to stromal HK cells in the absence and presence of ibrutinib treatment (12 h). (**d**) Colony formation assay showing enhanced clonogenic growth in IR MCL cells in the absence and presence of ibrutinib treatment. (**e**,**f**) Cell adhesion assay (**e**) and western blots (**f**) showing stable integrin β1 (CD29) knockdown with shRNAs significantly decreased rictor, pAKT, p4EBP and pS6K1 in IR MCL cells and significantly attenuated cell adhesion of parental (Sen) and IR MCL cells to HK stromal cells. (**g**) Co-immunoprecipitation revealed β1 and ILK form a complex in Jeko-1-IR and HBL-2-IR cells and β1 knockdown abolished β1/ILK complex formation. *indicates the specific band for integrin β1 (**h**) Co-immunoprecipitation showing ILK co-immunoprecipitates with rictor to form a more abundant complex in Jeko-1-IR and HBL-2-IR cells versus that shown in their parental (Sen) cells. (**i**) Co-immunoprecipitation showing suppression of β1/ILK co-immunoprecipite formation in parental (Sen) but not in IR MCL cells after ibrutinib treatment in HBL-2-IR and Jeko-1-IR cells. Scale bar, 10 μm. Results in **a**–**g** are representative of at least three independent experiments or means±s.d. from at least three biological replicates. For **a**–**f**, relative changes of proteins were measured by quantitative densitometry and indicated below each lane. See also Supplementary Figs 3, 8 for full gel scan of the WB.

We detected increased β1 expression in IR MCL cells compared with parental lines (Fig. 3a,b). Moreover, flow cytometry showed that β1 expression was further enhanced upon co-culture with stroma (Supplementary Fig. 3a). After ibrutinib exposure, β1 expression levels were reduced in parental/naïve, although levels remained unchanged after ibrutinib treatment in IR MCL cells (Fig. 3a,b). Adhesion assays and clonogenic growth experiments with and without ibrutinib treatment showed that IR MCL cells had a marked increase in adhesion to stromal cells and enhanced clonogenic growth in the presence of ibrutinib (Fig. 3c,d and Supplementary Fig. 3b). Furthermore, stable β1 knockdown dramatically attenuated cell adhesion to stromal cells, cell survival and clonogenic growth in IR cells (Fig. 3e,f and Supplementary Fig. 3c–e).

The interplay of persistent PI3K-AKT-mTORC activation and β1 expression were evaluated to gain insight into the underlying mechanism of PI3K-AKT-mTOR pathway activation in IR MCL cells. Indeed, mTORC2 signalling and mTORC1 downstream pS6K and p4EBP were significantly attenuated in stable β1 knockdown IR cell lines (Fig. 3f). Because β1 signals have been reported to be associated with ILK and ILK activation has been consistently identified in IR cells by ABPP (Fig. 2b–f), co-immunoprecipitation assay revealed that β1 immunoprecipitated with ILK in IR cells (Fig. 3g,h). In contrast, no β1-ILK complex formations were detected in β1 knockdown stable Jeko-1-IR and HBL2-IR cells. We also probed for co-precipitated ILK with rictor, an upstream activator of AKT activation, and identified ILK-rictor complexes in Jeko-1-IR and HBL-2-IR cells (Fig. 3h). These data indicate that β1 regulates mTORC2-AKT pathway activation via ILK-rictor complex formation. We also examined β1-ILK-rictor complex formation after ibrutinib treatment and observed persistent β-ILK-rictor complexes in IR cells but not in their parental cells (Fig. 3i). Together, these results demonstrate the reciprocal activation loop of PI3K-AKT-mTOR and β1 signalling, a central signalling hub for IR, resulting in enforced TME–lymphoma interactions and promoting MCL growth and drug resistance.

### Functional dissection of mTORC1 and mTORC2 in IR MCL cells

**Figure 4:**
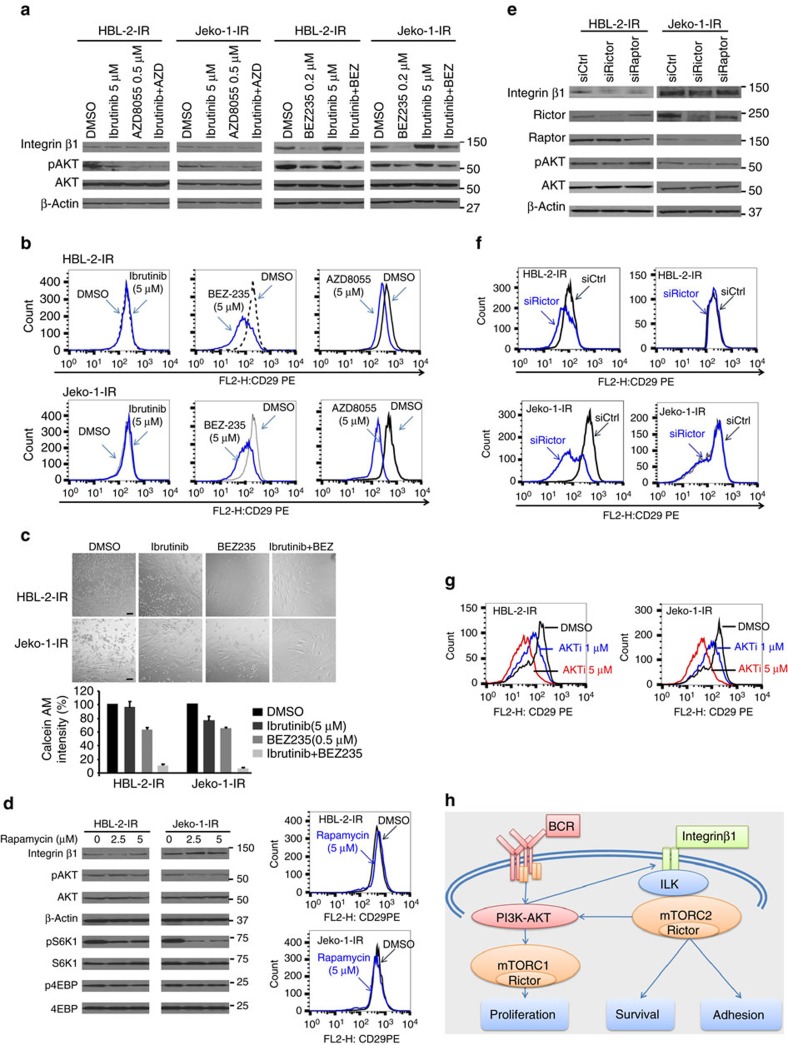
Functional dissection of mTORC1 and mTORC2 in cell growth and stroma/MCL interaction in acquired IR MCL cells. (**a**–**c**) Treatment with the PI3K/mTOR inhibitors BEZ235 and AZD8055, but not ibrutinib, significantly decreased AKT phosphorylation, β1 expression (**a**,**b**), and adhesion to HK stromal cells (**c**) in IR MCL cells. (**d**) Rapamycin inhibited mTORC1 activation, but had no effect on AKT activation and β1 expression in IR MCL cells. (**e**,**f**) Inhibition of mTORC2 by rictor knockdown using a pool of siRNAs decreased AKT activation, β1 expression and cell adhesion. In contrast, blocking mTOR1 by raptor knockdown with siRNA showed no changes in AKT activation and β1 expression in IR MCL cells. (**g**) Treatment with AKT inhibitor (AKTi, A674563, for 12 h) significantly attenuated β1 (CD29) expression in IR MCL cells. (**h**) Diagram of functional regulation of mTORC1 and mTORC2 in proliferation, AKT activation, β1 expression and cell adhesion in IR cells. Results in **a**–**g** are representative of at least three independent experiments or means±s.d. from at least three biological replicates. See also Supplementary Figs 4, 8 for full gel scan of the WB.

To functionally define the distinct role of mTORC1 and/or mTORC2 in MCL growth and cell adhesion to TME stroma, we used the dual PI3K-mTOR1/2 inhibitor BEZ235 and the dual mTOR1/2 inhibitor AZD8055. BEZ235 and AZD8055 decreased AKT phosphorylation, β1 expression and adhesion to stroma in Jeko-1-IR and HBL-2-IR cells (Fig. 4a–c, Supplementary Fig. 4a). BEZ235 induced G1 arrest in Jeko-1-IR and HBL-2-IR cells (Supplementary Fig. 4b). In contrast, rapamycin, an allosteric inhibitor of mTORC1, induced cell cycle G1 arrest, attenuated activation of mTORC1 downstream proteins such as S6K1, but had no effect on AKT activation, β1 expression or cell adhesion (Fig. 4d and Supplementary Fig. 4c). Consistent with these data, we observed that blocking mTORC2 by rictor knockdown using siRNAs decreased β1 and pAKT levels and decreased cell adhesion. However, blocking mTORC1 by raptor knockdown using siRNAs showed no changes in β1 expression and cell adhesion (Fig. 4e,f, Supplementary Fig. 4d). In line with these data, inhibition of AKT attenuated β1 expression and cell adhesion (Fig. 4g, Supplementary Fig. 4e,f). Collectively, the functional regulation of the PI3K-AKT-mTORC1 pathway is essential for cell growth and the mTORC2-AKT pathway contributes to β1 expression, adhesion and proliferation in IR MCL cells (summarized in Fig. 4h).

### Unification of TME-mediated and acquired IR in MCL

**Figure 5:**
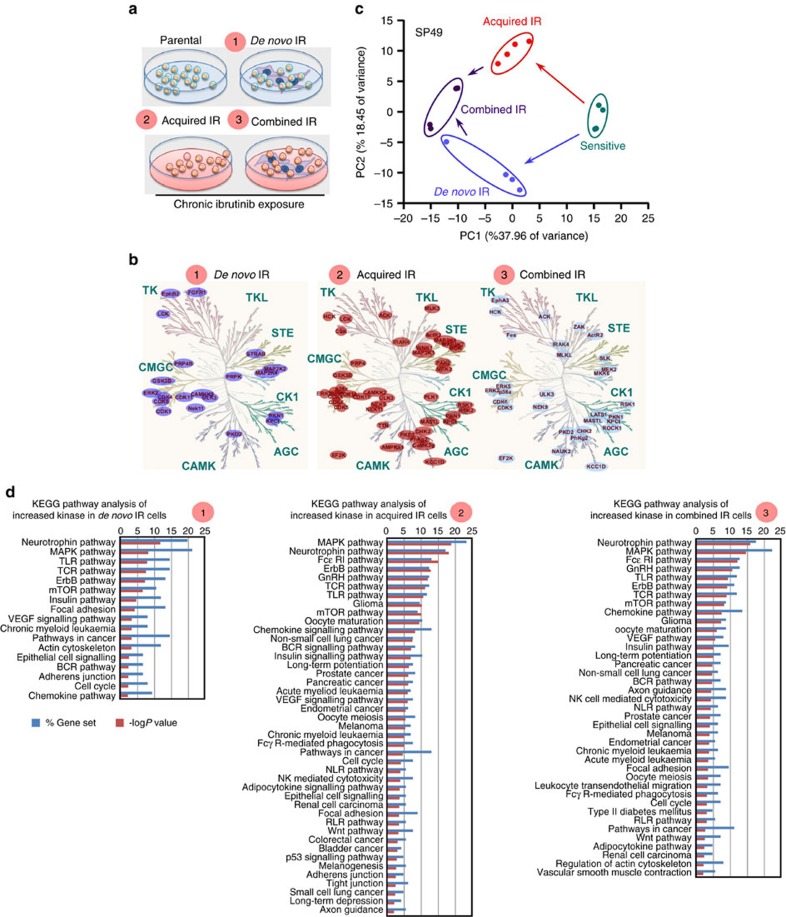
Functional coordination between TME-mediated (*de novo*) and acquired IR. (**a**) Diagram of three types of drug resistance mechanisms for kinase activity profile: parental cells after co-culture with stromal (HK) cells (*de novo* DR), IR cells after chronic ibrutinib exposure in cell suspension (acquired DR) and IR cells selected with drug (chronic ibrutinib exposure) in the presence of stroma (combined DR). Parental cells in suspension are as reference. (**b**) Significant increased kinase ATP-binding changes in *de novo* DR cells (left), acquired DR cells (middle) and combined DR (right). Parental cells maintained in suspension were used as the control reference population (Jeko-1, HBL-2 and SP49). Kinome trees reproduced courtesy of Cell Signaling Technology. (**c**) Principal component analysis (PCA) of kinomes of parental/sensitive, *de novo* IR (TME-mediated), acquired IR and IR selected while adherent to TME stroma (combined IR) cells, which correlate with the first two principal components. (**d**) KEGG pathway enrichment analysis revealed highly significant pathways (−log *P* value>2) in the three types of IR kinome. Parental cells maintained in suspension were used as the control reference population. There is a greater overlap between acquired IR and combined IR relative to *de novo* IR. See also Supplementary Fig. 5.

With our demonstration that IR correlated with increased β1 expression, we anticipated that this would lead to a greater potential for IR cells to interact with TME compared with naïve cells in the presence of ibrutinib. Using the three naïve/IR cell line pairs, we sought to address the question of how cell adhesion influences acquired drug resistance. We elucidated kinome network of (1) *de novo* (TME-mediated), (2) acquired drug resistance and (3) IR selected while adherent to TME stroma (combined IR) (Fig. 5a). Our ABPP analysis detected 220 kinases, including 24 kinases with more ATP binding than when these cells were adhered to stromal HK cells (Fig. 5b). In addition, we performed and compared the HK-induced kinome alteration with bone marrow stroma HS-5-induced kinome changes and found significant overlap between these two conditions in all three cell lines (Supplementary Fig. 5a), supporting that HK and HS-5 mediated common kinase signalling at least to some degree. Furthermore, acquired IR was associated with 205 kinase alterations, including 40 kinases with greater ATP binding (Fig. 5b). IR selected in the presence of stroma designated as IR-TME (Fig. 5b) demonstrated 210 kinases, including 33 with greater ATP binding (Fig. 5b, right). Integrated principal component analysis showed that all three types of drug resistance clustered, respectively, and distant from parental cells, and combined IR shared kinase signature with both *de novo* and acquired IR (Fig. 5c, Supplementary Fig. 5b). With further pathway analysis by KEGG, we found that all three conditions demonstrated a greater overlap of kinome reprogramming and pathways in IR and IR-TME relative to those of *de novo* TME-mediated drug resistance (Fig. 5d, Supplementary Fig. 5b). Similar to acquired IR cells, β1 and PI3K/AKT signalling were comparably activated and maintained at higher levels after ibrutinib treatment in these IR-TME cells (Supplementary Fig. 5c). We also observed that these cells have increased growth potential, are more adhesive and conferred more clonogenic growth (Supplementary Fig. 5d–f) similar to that shown in IR phenotypes. Overall, these data provide the biological significance of the kinome alterations, and these two forms of resistance interact in a unified way to initiate and amplify the drug resistant phenotypes.

### Ibrutinib resistance in *in vivo* murine model and primary MCL

We applied parental and IR MCL cells to *in vivo* xenograft models to validate (1) whether TME stroma promotes tumour progression in both parental and IR MCL cells, (2) whether IR MCL cells conferred greater tumour-forming potential and (3) whether PI3K-AKT-mTOR activation was sustained and β1 was upregulated. Parental and IR HBL-2 cells with or without HK stromal cells were subcutaneously injected into the lower flank of NOD/SCID mice, and tumour volume was assessed. Consistent with previous results, a more robust growth of tumours was observed in mice receiving HK and HBL-2 cells than in mice injected with HBL-2 cells alone^2^,^27^.

**Figure 6:**
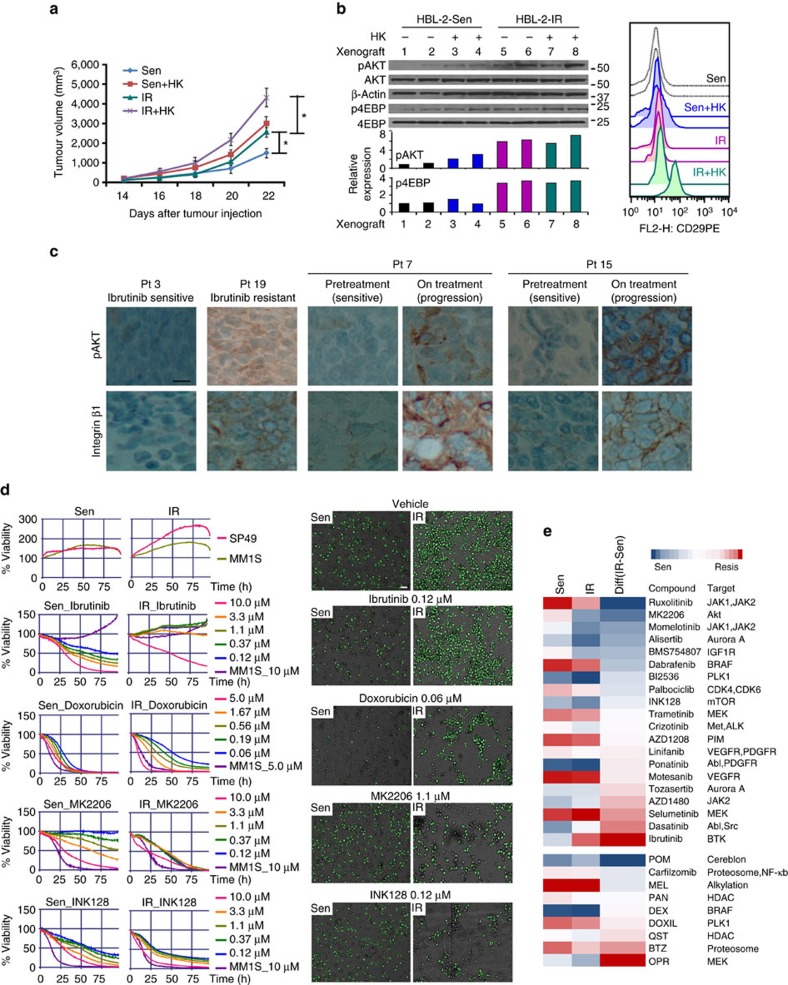
IR mechanism sustained in *in vivo* xenograft model, *ex vivo* primary MCL specimens and recapitulated in drug screening assay. (**a**) Xenograft experiments showed that HK cells enhanced tumour formation of both parental (Sen) and IR MCL cells (HBL-2). IR MCL cells had more robust tumour formation *in vivo* compared with parental cells. *P<0.05, *n*=5 per cohort (Student's *t*-test). (**b**) Immunoblot and flow cytometry conducted on MCL samples from NOD/SCID mice injected with HBL-2 cells and IR HBL-2 with and without co-injection of HK cells of cohorts as described in **a**. 1–8 represent eight xenografts, with relative expression of pAKT and p-4EBP were quantitated by quantitative densitometry and shown in coloured bar graph. (**c**) Immunohistochemistry stains showing elevated AKT activation (**c**) and β1 expression (**d**) in IR MCL patient samples when compared with ibrutinib-sensitive samples or samples on the progression after ibrutinib treatments. Scale bar, 10 μm. (**d**) A cell-based drug screening assay was used to measure cell growth and chemosensitivity to indicated chemical agents in a reconstructed TME. Parental and IR cells (SP49-Sen and SP49-IR) were seeded in 384-well plates of reconstructed bone marrow, including high physiological cell densities (1–10 × 10^6^ cells per ml), extracellular matrix (collagen 1) and human bone marrow derived stromal cells (BMSC). A panel of drugs at five different concentrations was added to the media, and plates were continuously imaged for 96 h using a digital image analysis algorithm to identify viable cells based on membrane motion (pseudo-coloured in green). Changes in viability are quantified by area under curve (AUC). Left, cell growth and viability of sensitive (Sen) and IR MCL cells was measured with no treatment, treatment with ibrutinib, doxorubicin, MK2206 or INK128; right, images at 48 h in these conditions. Negative controls (no drug) were included, as well as positive controls for each drug (cell line MM1.S at highest drug concentration). Scale bar, 30 μm. (**e**) Heat map showing chemosensitivity of parental (Sen) and IR cells (Resis) to 31 agents including protein kinase inhibitors, inhibitors of enzymatic processes and chemotherapeutic agents. See also Supplementary Figs 6, 8 for full gel scan of the WB.

Given our previous demonstration that HK cells alone could not induce lymphoma formation and that very small to minimal amounts of HK cell were present even in co-injection-induced lymphomas, we concluded that the robust growth of tumours is attributed to MCL growth and proliferation^2^. Importantly, compared with parental sensitive cells, the MCL tumour volume derived from IR cells demonstrated greater tumour volume both with and without concomitant stromal cells (Fig. 6a), suggesting greater tumour-forming potential. Mechanistically, western blot and flow cytometry revealed higher β1 expression, AKT/4EBP phosphorylation in xenografted MCL tumors from mice receiving HK and HBL-2 cells and in xenografted MCL tumors in mice receiving IR HBL-2 cells compared with those receiving HBL-2 cells only or parental cells (Fig. 6b). Immunohistochemical staining of primary ibrutinib-sensitive and IR patient MCL samples revealed increased AKT activation and β1 expression in IR MCL cells (Fig. 6c, Supplementary Fig. 6a). We observed significantly increased β1 expression levels in six of seven IR MCL samples, whereas only one of eight ibrutinib-sensitive MCL samples revealed a moderate β1 staining. Moreover, two patient samples that initially responded but ultimately progressed were from biopsies at the time of initial diagnosis and at relapse on ibrutinib treatment (Fig. 6c).

Next, we capitalized on a novel *ex vivo* organotypic live imaging cell-based platform to assess drug response in parental and IR cells in reconstructed TME^28^,^29^. In this system, MCL cells (SP49) of parental and IR were seeded in a 384-well plate previously coated with human-derived stroma cells and collagen-1, and drug screen was performed using 31 agents, including protein kinase inhibitors, inhibitors of deacetylase, proteasome inhibitors and chemotherapeutic agents. As shown in Fig. 6d, IR cells conferred greater growth potential and became more adherent compared with the parental cell line. Increased viability in the presence of ibrutinib was also noted in IR cells. Treatment with doxorubicin resulted in more viable IR cells and/or stroma-mediated drug resistance. Critically, AKT and mTOR inhibitors demonstrated more dramatic attenuations of cell viability in IR cells versus naïve cells, consistent with our hypothesis that this pathway serves as a key compensatory mechanism in IR. Importantly, the *ex vivo* screen also demonstrated that protein kinase inhibitors targeting JAK1, JAK2, aurora A, IGF1R and PLK suppressed cell growth of IR MCL cells (Fig. 6e). Of note, these pathways/kinases were also identified through ABPP in IR MCL cells (Fig. 2); thus a functional proof of principal for adaptive changes was observed in IR cell kinome.

### Novel therapeutics in murine and patient-derived xenografts

**Figure 7:**
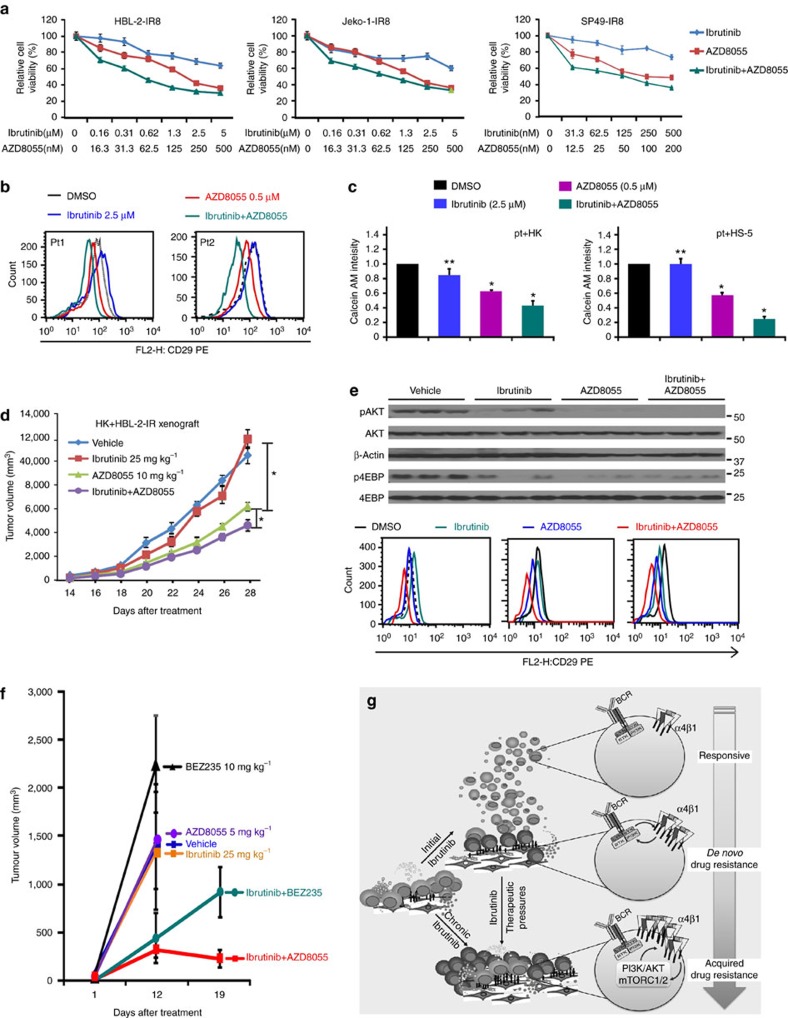
Inhibition of the PI3K-AKT-mTOR pathway overcomes IR in *ex vivo* and *in vivo* using patient derived xenografts (PDX) model. (**a**–**c**) AZD8055 treatment in combination with ibrutinib more substantially inhibited cell viability measured by CCK8 assay in IR MCL cells (**a**), more substantially inhibited β1 expression (**b**) in two IR primary samples and cell adhesion (**c**) in four IR primary MCL patient samples. *P<0.05 and **P≥0.05 (Student's *t*-test). (**d**,**e**) AZD8055 treatment led to a reduction in tumour growth that was enhanced in combination with ibrutinib (co-treatment). (**d**) the co-treatment of AZD8055 and ibrutinib markedly reduced pAKT, p4EBP and CD29 levels (**e**) in murine xenografts as indicated. (**f**) Combinatorial treatments of BEZ235 or AZD8055 with ibrutinib induced more dramatic anti-MCL activity in a patient-derived xenograft from IR MCL (PDX). The NSG mice bearing the PDXs were randomly divided into six groups (5 per group), with growing tumours subsequently treated with solvent (equal volume of vehicle, blue), ibrutinib 25 mg kg^−1^ alone (orange), AZD8055 5 mg kg^−1^ alone (purple), BEZ235 10 mg kg^−1^ alone (green), ibrutinib 25 mg kg^−1^ with AZD8055 5 mg kg^−1^ (red), or ibrutinib 25 mg kg^−1^ with BEZ235 10 mg kg^−1^ (green) all by oral gavage daily. Tumour burden was assessed by tumour volume measurements at days 1, 12 and 19 after treatment. (**g**) A simplified IR model showing enforced interaction of the MCL-stromal cells and IR development under ibrutinib treatment. β1 contributed to the PI3K-AKT-mTOR1 activation through forming complex with ILK and mTORC2 and that PI3K-AKT, in turn, induced β1 expression, thereby generating a positive feedback loop, ensuring high level and sustained TME–lymphoma interaction and allowing cells to acquire a more permanent and complex drug resistance phenotype in MCL cells. Results in **a**,**c** are shown as mean+s.d. from at least three biological replicates. See also Supplementary Figs 7, 8 for full gel scan of the WB.

To further validate the compensatory role of PI3K-AKT-mTOR signalling in IR, we turned to the three pairs of parental and IR MCL lines. When BEZ235 or AZD8055 was combined with ibrutinib, cell survival, β1 expression, cell adhesion and clonogenic growth were substantially inhibited in all IR MCL lines and in patient samples of acquired IR MCL (Fig. 7a–c, Supplementary Fig. 7a,b). As shown in Fig. 7c, AZD8055 or AZD8055 plus ibrutinib, but not ibrutinib alone, induced significant changes in cell adhesion to stroma in IR MCL samples. Persistent β1 expression and cell adhesion to stroma despite ibrutinib treatment were observed in these patient samples, with BEZ235 and AZD8055 in combination with ibrutinib more significantly reducing β1 expression, AKT activation and cell adhesion to stroma (Fig. 7b,c, Supplementary Fig. 7c). We further administered AZD8055 in NOD/SCID mouse for its anti-lymphoma activity in combination with ibrutinib by using HBL-2-IR MCL cells and HK stroma cells. As expected, HBL-2-IR was resistant to ibrutinib treatment; however, AZD8055 induced remarkable inhibition of MCL growth and in combination with ibrutinib further suppressed lymphoma formation. ADZ8055 in combination with ibrutinib markedly reduced pAKT, pS6K1, p4EBP and β1 expression levels and reduced cell adhesion to HK cells in these xenograft tumour cells (Fig. 7d,e, Supplementary Fig. 7d).

To further examine our hypothesis, we used our previously described MCL PDX model to test response of IR cell lines to ibrutinib and combination treatment. As shown by Wang *et al*.^30^, a SCID-hu mouse model developed for myeloma allowed primary human MCL cells to be injected directly into the microenvironment of human fetal bone in SCID mice. In the SCID-hu mice, the bone implants vascularized and were histologically similar to bone marrow, providing a TME for tumour growth with lymphoma manifestations such as increased serum human β2 microglobulin levels. We found that NOD/SCID IL-2Rγ (NSG) mice are a more suitable strain for PDX propagation for first generation of PDXs in SCID-hu mice. For this experiment, MCL cells isolated from an abdominal mass of a 74-year-old female patient who progressed after ibrutinib treatment were injected directly into the human fetal bone of SCID mice. Four to eight weeks after inoculation, a large tumour mass was observed around the implanted human bone, spleen and abdominal lymph node. The PDXs established in the SCID-hu model were then transferred from the SCID-hu mice into NSG mice, and we randomly divided the mice bearing the cells from the same patient into treatment groups (5 mice per group). Histological examination, CD20 and cyclin D1 analyses, and flow cytometry confirmed MCL diagnosis, with tumours comparable to the original diagnosed MCL (Supplementary Fig. 7e).

As shown in Fig. 7f, NSG mice bearing the PDXs were treated with one of the following six conditions: vehicle, ibrutinib alone, AZD8055 alone, BEZ235 alone, ibrutinib with AZD8055 and ibrutinib with BEZ235 (all by daily oral gavage). Tumour volume was assessed on days 1, 12 and 19 after treatment. Consistent with the IR nature, the tumours in all mice treated with single-agent ibrutinib grew rapidly despite drug treatment, and tumour volumes were unchanged or even increased with low-dose AZD8055 or BEZ235 alone compared with vehicle (Fig. 7f). In contrast, tumour volumes were significantly decreased in the two-drug combination groups of AZD8055 or BEZ235 plus ibrutinib compared with control or ibrutinib monotherapy (Fig. 7f).

## Discussion

Acquired drug resistance has traditionally been studied by chronically exposing cancer cell lines *in vitro* to increasing doses of drug. These drug-resistant models have been instrumental in identifying mechanisms that modulate drug response and, in some cases, have aided in the identification of drug targets^31^. However, these models have failed to account for the TME in the emergence of the drug-resistant phenotype. Here, we combined lymphoma TME models, using functional and chemical proteomics approaches, to define the key protein kinase reprogramming and a central signalling hub for TME-induced- and acquired IR in MCL.

PI3K-AKT pathway activation has been identified as an important drug resistance mechanism in many tumours. However, our study uniquely characterized the development of acquired IR in the context of TME and unified TME-mediated and acquired IR, providing a mechanistic link between TME and acquired IR. Our data indicated that their interplay and cooperative function are critical for promoting tumour growth, IR and MCL progression. In addition, we identified integrin β1-ILK signalling as the underlying mechanism of not only PI3K-AKT activation in MCL IR but also TME in facilitating acquired IR resistance. More importantly, our functional and chemical proteomic results were independently validated by cell-based drug screen experiments in the setting of TME, implicating this drug screen assay as an innovative platform to help with design of effective individualized combination therapies to overcome IR and disable aggressive lymphomas. A simplified model showing the enforced interaction of the MCL-TME and development of IR is illustrated in Fig. 7g. As shown here, the TME–lymphoma interaction allows MCL cells to survive initial drug exposure and eventually acquire a complex array of acquired drug resistance mechanisms through kinome reprogramming.

We compared functional resistance mechanisms and corresponding kinase activation profiles associated with *de novo* TME-mediated and acquired resistance mechanisms. When MCL cell lines selected in the absence of stroma for acquired IR were compared with parental MCL cell lines after adhesion (co-culture) to the stroma (*de novo*, EMDR), we found that acquired IR mechanism was more complex than that of EMDR. The complex changes in IR MCL cell kinome could contribute to the observed phenotypic alterations. The kinomic changes account for increased proliferation and drug resistance in acquired resistant MCL cells, whereas activation of adhesion/integrin β1 signalling pathways contributed to enhanced TME–MCL cell interaction and amplified EMDR. Despite differences in the models for acquired and *de novo* drug resistance, we observed a significant overlap in kinase changes, supporting that EMDR is essential for initial survival, priming MCL cells for the acquisition of stable drug resistance. In line with this, our *in vivo* xenograft model revealed that TME stroma cells promote MCL growth and that IR MCL cells confer an advantage in stroma interaction, drug resistance and clonogenic growth ability. The adaptive kinome reprogramming and potential targets were further evidenced by an independent *ex vivo* drug screening that quantified drug sensitivity in a reconstructed TME. Furthermore, when selecting IR to chronic ibrutinib exposure in MCL cells adherent to stroma and developing IR MCL lines in the setting of TME (IR-TME), we observed that the kinome profile of cells from ‘combined selection' are more closely related to the cells with acquired resistance than cells selected by *de novo* resistance in co-culture with stroma. Comparable changes in β1-ILK expression, PI3K-AKT-mTOR pathway activation, enhanced cell adhesion and growth in MCL cells were detected with both conditions. These results highlight that development of IR represents a multiple stepwise process requiring a complex interplay between TME signals, gene and protein adaptation, and selection pressures, with ILK and PI3K-AKT-mTOR being potential target for overcoming IR.

Initially, cell–cell or cell–TME interactions attract MCL cells to the stromal niche, conferring MCL BCR signal for MCL survival. When therapeutic stress was applied, a series of cell responses occurred in both MCL cells and stromal cells, creating a positive feedback loop that amplified the pro-survival and anti-apoptotic signals, with eventual TME-independent acquired resistance dominating the late stage, although TME still physically or biochemically influenced the drug resistance phenotype. Together, our data support that the drug-resistant population presents a kinase activity pattern different from the one seen in the original naïve tumour. This is because *de novo* and/or acquired drug resistance mechanisms may drive clonal evolution before, during and after therapies.

Our results are consistent with the complex nature of resistance mechanisms. Resistance is often ascribed to either resistant mutations such as BTK and PLCγ2 reported in IR MCL and chronic lymphocytic leukaemia or adaptive mechanisms, including amplification of alternative signalling pathways and altered cell states^19^,^20^. Given the complex heterogeneous nature of MCL and its TME, resistance to anti-cancer therapies probably stems from pre-existing clonal heterogeneity and clonal evolution, resulting from a combination of mutations or alteration of genetic and epigenetic determinants as part of adaptive responses to therapy and interplay with TME^32^,^33^,^34^. Our data further highlight that tumour cell heterogeneity and clonal evolution in response to the selective pressure of a toxic agent usually prevents long-term efficacy of any monotherapy. Advances in proteomics techniques have now allowed the simultaneous quantification of several thousand proteins, which can be used to derive signalling networks from large-scale data sets^35^,^36^,^37^.

In our study, we used ABPP and defined a kinase signalling network in parental and IR MCL cells and the adaptive rewiring in response to ibrutinib in *de novo* TME and acquired drug resistance. The remodelling of kinase networks in IR cells produced patterns of signalling activity linked to their evolved phenotypes. Given the myriad of therapeutic resistance mechanisms, including point mutations in known targets, network-wide approaches are crucial to improving our understanding of altered survival and drug resistance as a consequence of reprogramming and ultimately determining the success or failure of therapeutic agents. Thus, when considering the use of targeted therapies in cancer, it is necessary to understand and integrate the multiple tumour cell adaptation mechanisms to a given therapy to better design a combination of therapies with optimal anti-tumour effects. The identification of ‘common signalling nodes' or drivers for resistant clonal evolution in multiple altered signalling pathways that confer resistance is warranted. Our findings suggest a more complex network to stable genetically conferred resistance to cancer drugs than the specific drug resistance mutations. Such mutations could arise spontaneously at low frequency before drug treatment and are selected during treatment. However, our observations implicate a multistep stepwise process mediated by TME and tumour interaction is not incompatible with ‘pre-existing' resistance-conferring mutations. Because of the dynamics and complexity of tumour evolution and often lack of correlation between mutations with therapy response, our cell-based drug screen in the setting of TME can provide a promising tool for effective individualized therapy against MCL and other B-cell lymphomas. The continued application of this approach in combination with genomes, functional transcriptome and proteomes to primary and recurrent tumours will identify novel mechanisms driving tumour progression and therapeutic resistance, facilitating the identification of optimal combinatorial therapeutic strategies. The inhibition of these pathways and resistance networks could be a more efficient way to overcome drug resistance and improve current therapies for patients with MCL and other B-cell malignancies.

## Methods

### Activity-based protein profiling

Briefly, cell pellets were sonicated in IP/lysis buffer, desalted with Zeba spin desalting column and followed by incubation with 10 μM desthiobiotin-ATP probes at room temperature for 10 min. The labelled proteins were reduced, alkylated and trypsin digested at 37 °C for 2 h. The labelled peptides were purified with high capacity streptavidin agarose resin, washed, eluted and subjected to LC-MS/MS for quantification. The procedure of ABPP and measurement of secretome are detailed in Supplementary Method.

### Imaging- and cell-based drug screening assay

Parental or IR cells (SP49 and SP49-IR) were seeded in multi-well plates with previously established human-derived stroma and collagen-α to a total volume of 8 μl. Each well was filled with 80 μl of RPMI 1640 media supplemented with FBS (10%, heat inactivated), antibiotics (1% penicillin/streptomycin) and left overnight for adhesion of stroma. The next day drugs were added using a robotic plate handler, so that every drug was tested at five concentrations (1:3 serial dilution) and two replicates. Negative controls (no drug) were included, as well as positive controls for each drug (cell line MM1.S at highest drug concentration). Plates were placed in a motorized stage microscope (EVOS Auto FL, Life Technologies) equipped with an incubator and maintained at 5% CO_2_ and 37 °C. Each well was imaged every 30 min for a total duration of 4 days (96 h). To determine changes in viability of each well longitudinally across the 96 h interval, a digital image analysis algorithm was used. In summary, this algorithm computes differences in sequential images and identifies as live cells those with continuous membrane deformations (pseudo-coloured in green) resulting from the interaction with the surrounding matrix. These interactions cease upon cell death. By applying this operation to all 192 images acquired for each well, it is possible to quantify non-destructively and without the need of separation between stroma and lymphoma cells, the effect of drugs as a function of concentration and exposure. Changes in viability are quantified by area under curve (AUC)^28^.

### Sanger sequencing

Genomic DNAs were isolated from MCL cell lines using the DNeasy blood and tissue kit (Qiagen). The following primers were used to amplify the DNA regions encoding either the wild type or the C481S and R665W mutations in BTK and PLCG2 respectively (Life Technology)

BTK: 5′-AGTTGTATGGCGTCTGCACCAA-3′ (forward)

5′-AGGTCTCGGTGAAGGAACTGCT-3′ (reverse).

PLCG2: 5′-GGTCCAAGGCTTTCAGAAACCCC-3′ (forward)

5′-GAAGGACAGGGTGTAGTCATTGGGG-3′ (reverse).

DNAs were amplified with the Platinum Taq DNA polymerase (Life Technology) and PCR products were purified with the QIAquick PCR purification kit (Qiagen). Sanger sequencing was performed at the Institute of Biotechnology of Cornell University.

### Patient-derived xenograft model

Primary MCL cells were isolated from lymph node of patients and injected into human bone chips and performed as previously described^30^. For a more detailed description about generation and features of PDX model and clinical characterization of IR patient used for PDX, see the Supplementary Method. Cell assay, western blotting (uncropped scans of the blots are also available in the Supplementary Information), mouse model and statistical details are also provided in Supplementary Method.

### Data availability

ABPP raw data are available via ProteomeXchange with identifier PXD005734, Kinase peptide lists and intensity are in available in Supplementary Data 1. Other data that support the findings of this study are available from the authors on reasonable request; see author contributions for specific data sets.

## Additional information

**How to cite this article:** Zhao, X. *et al*. Unification of *de novo* and acquired ibrutinib resistance in mantle cell lymphoma. *Nat. Commun.* **8,** 14920 doi: 10.1038/ncomms14920 (2017).

**Publisher's note:** Springer Nature remains neutral with regard to jurisdictional claims in published maps and institutional affiliations.

## Supplementary Material

Supplementary Information Supplementary Figures, Supplementary Methods and Supplementary References

Supplementary Data 1 Kinase list of Ibrutinib Sensitive and Resistant from ABPP experiment*.
