# Supplementary material for: Unification of de novo and acquired ibrutinib resistance in mantle cell lymphoma
Source: Nat Commun. 2017 Apr 18;8:14920. doi: 10.1038/ncomms14920 (PMC5399304; doi:10.1038/ncomms14920)
Supplement: Supplementary Information — Supplementary Figures, Supplementary Methods and Supplementary References [file ncomms14920-s1.pdf]

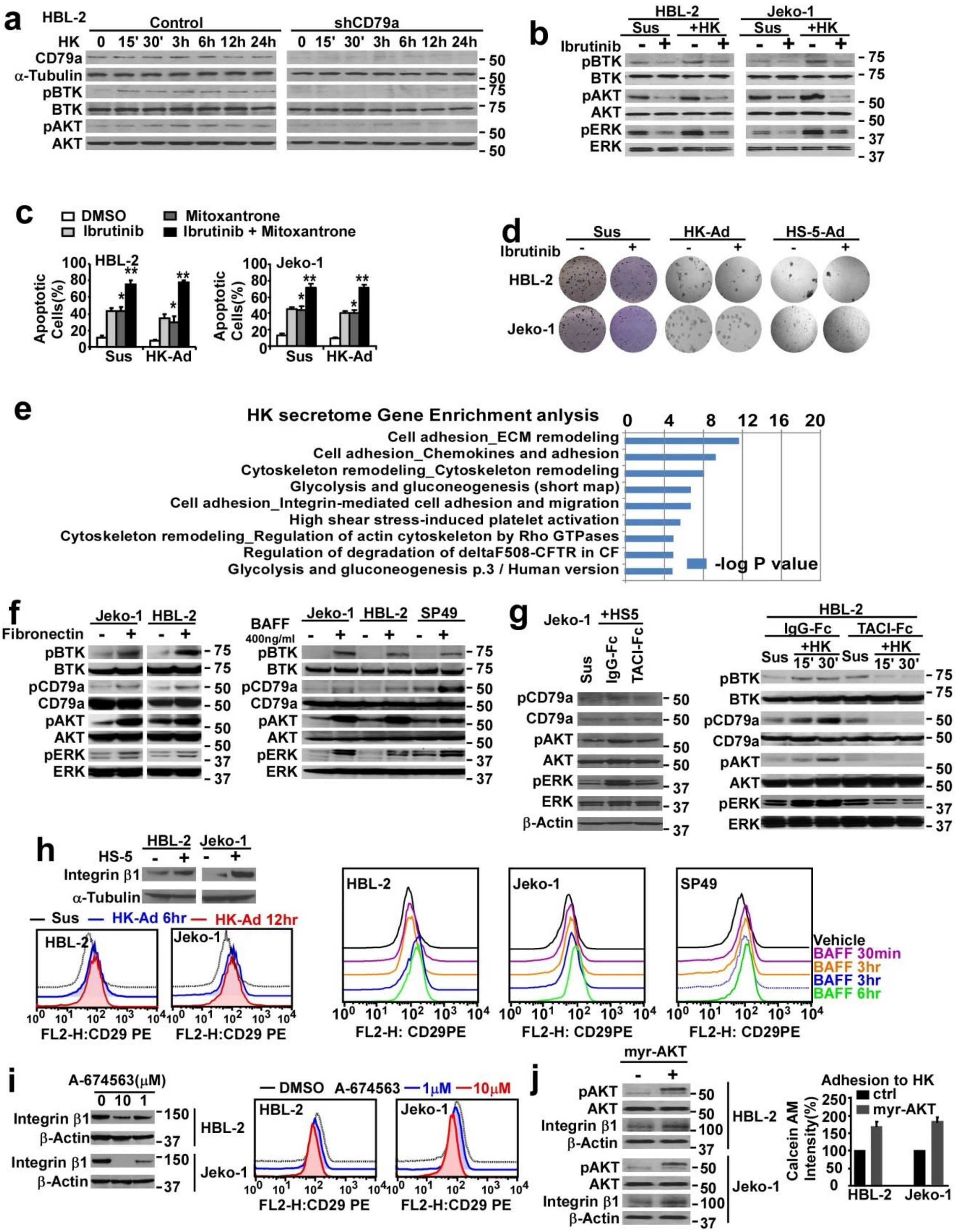

a
HBL-2
Control
shCD79a
HK
0 15' 30' 3h 6h 12h 24h
CD79a
α-Tubulin
pBTK
BTK
pAKT
AKT
b
HBL-2
Jeko-1
Sus
+HK
Ibrutinib
pBTK
BTK
pAKT
AKT
pERK
ERK
c
DMSO
Mitoxantrone
Ibrutinib
Ibrutinib + Mitoxantrone
Apoptotic Cells(%)
Sus
HK-Ad
d
Sus
HK-Ad
HS-5-Ad
Ibrutinib
HBL-2
Jeko-1
e
HK secretome Gene Enrichment anlysis
Cell adhesion_ECM remodeling
Cell adhesion_Chemokines and adhesion
Cytoskeleton remodeling_Cytoskeleton remodeling
Glycolysis and gluconeogenesis (short map)
Cell adhesion_Integrin-mediated cell adhesion and migration
High shear stress-induced platelet activation
Cytoskeleton remodeling_Regulation of actin cytoskeleton by Rho GTPases
Regulation of degradation of deltaF508-CFTR in CF
Glycolysis and gluconeogenesis p.3 / Human version
-log P value
f
Fibronectin
BAFF 400ng/ml
SP49
pCD79a
g
+HS5
IgG-Fc
TACI-Fc
β-Actin
h
HS-5
Integrin β1
Sus
HK-Ad 6hr
HK-Ad 12hr
FL2-H:CD29 PE
Vehicle
BAFF 30min
BAFF 3hr
BAFF 3hr
BAFF 6hr
FL2-H: CD29PE
i
A-674563(μM)
DMSO
A-674563 1μM 10μM
j
myr-AKT
Adhesion to HK
ctrl
myr-AKT
Calcein AM Intensity(%)

Supplementary Figure 1. **B-Cell Receptor (BCR) signaling is a central "outside-in" and "inside-out" signaling hub for stroma-lymphoma interaction and stroma (TME)-mediated drug resistance and clonogenic growth in MCL cell lines and primary samples. (a).** CD79a knockdown by shRNA abolished HK induced BCR signaling activation in HBL-2 cells. Phosphorylation of BTK and AKT were detected by western blot. **(b).** Inhibition of BCR signaling using BTK inhibitor, ibrutinib, attenuated stroma-induced BTK, AKT and ERK phosphorylation in HBL-2 and Jeko-1 cells. **(c).** Ibrutinib induced cell apoptosis, overcame stroma-mediated drug resistance, and enhanced mitoxantrone (0.5 μM) induced apoptosis in HBL-2 and Jeko-1 cells. **(d).** Ibrutinib inhibited clonogenic growth with or without stroma co-culture. **(e).** Enrichment analysis of HK supernatant protein pathways. **(f).** Fibronectin (FN) and BAFF (400ng/ml) induced BCR signaling activation in MCL (Jeko-1, HBL-2, SP-49) lines. MCL cells were incubated with FN-coated plate for 30 min or treated with BAFF for 30 min, and the level and phosphorylation of CD79a, BTK, AKT and ERK were detected by western blot. The relative changes of proteins were measured by quantitative densitometry and indicated below each lane. **(g).** BAFF inhibition by soluble TACI-Fc (20ng/ml) suppressed stroma cell (HK or HS-5) induced BCR signaling in MCL cell lines. MCL cells were incubated with HK and either IgG-Fc or TACI-Fc for 30 min and the level and phosphorylation of CD79a, BTK, AKT and ERK were detected by western blot. **(h).** Co-culture with stromal cells HK or HS-5 and BAFF treatment up-regulated integrin β1 expression in HBL-2, Jeko-1 and SP49 cells detected by Western Blot and Flow cytometry. (HK-Ad, co-culture with HK; HS-5-Ad, coculture with HS-5). **(i-j).** AKT inhibitor A-674563 decreased integrin β1 expression in HBL-2 and Jeko-1 cells detected by Western Blot and Flow cytometry and constitutive activation of AKT by myr-AKT plasmid transfection increased cell adhesion to HK cell in HBL-2 and Jeko-1 cells. Results in a through i are representatives or mean ± SD from at least three biological replicates.

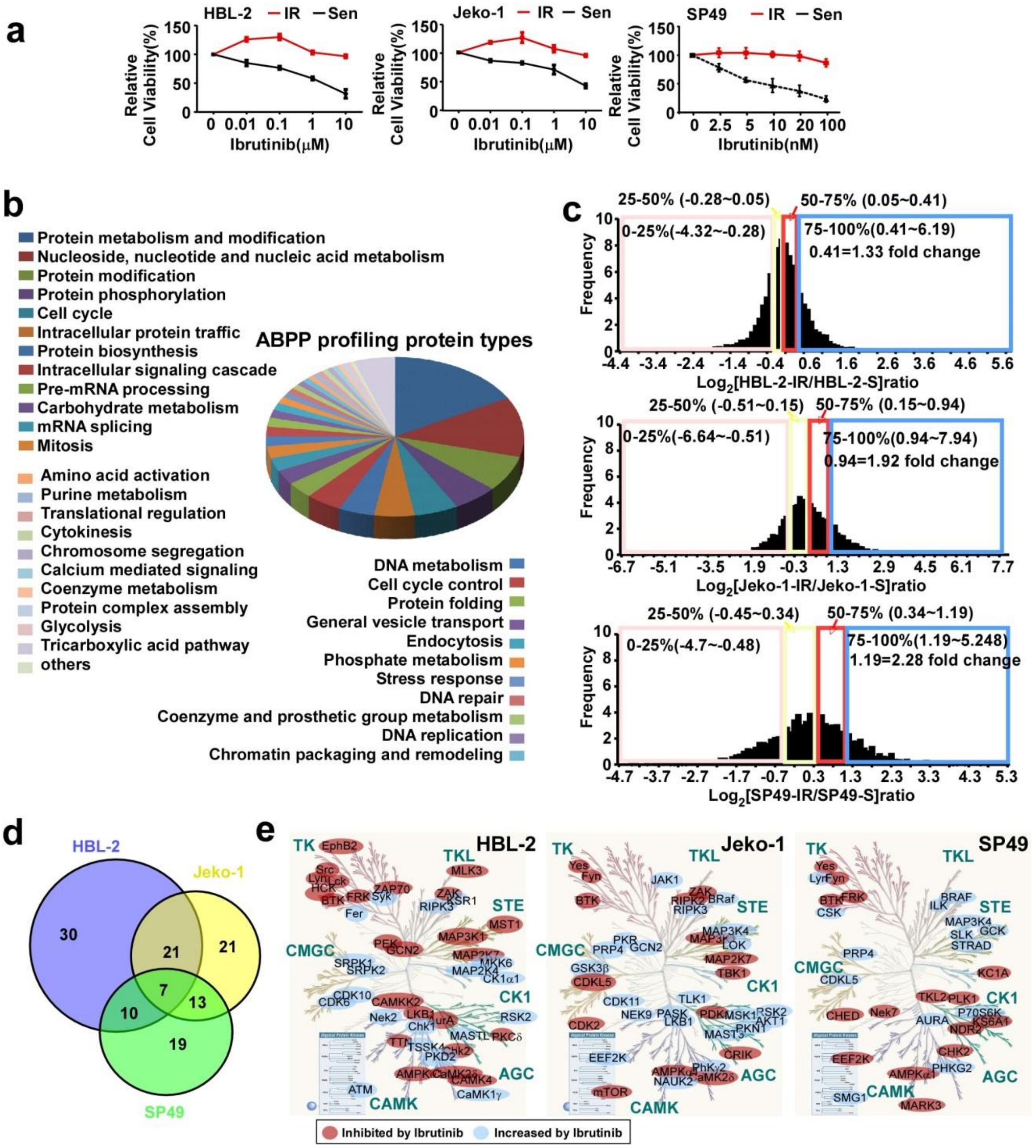

Supplementary Figure 2. Chronic ibrutinib treatment leads to adaptive kinome reprogramming and acquired ibrutinib-resistant in MCL cells. (a). Three independent IR MCL cell lines were generated by growing Jeko-1, HBL-2 and SP49 cells in increasing concentrations of ibrutinib over a period of six months. Cell viability assay of 3 pairs of parental (Sen) and IR cells performed by CCK8 assay. **(b).** Go Enrichment analysis of ABPP profiling using the PANTHER classification system[1]. Pie chart is protein classes for ABPP profiling identified in 3 pairs of IR and parental cells. **(c)**. Fold-change distribution of protein kinase comparing IR to parental cells. The distribution was divided into four quantiles as follows: high relative expression in IR cells (75%-100%), modestly up-regulated in IR cells (50-75%), modestly down-regulated in IR cells (25%-50%), low relative expression IR cells (0-25%). The fold changes of these categories were indicated at the top of the panel. The fold changes of 1.35 for HBL-2, 1.92 for

Jeko-1 and 2.28 for SP-49 cells were selected as cut-off values as these values defined the top quantile changes within each cell line. **(d).** Venn diagram shows the overlap of increased kinase activities among three IR MCL cells. **(e).** Significant kinase ATP-binding changes after 6 hr ibrutinib treatment in three parental MCL cell lines (Jeko-1, HBL-2, SP49). Kinome trees reproduced courtesy of Cell Signaling Technology.

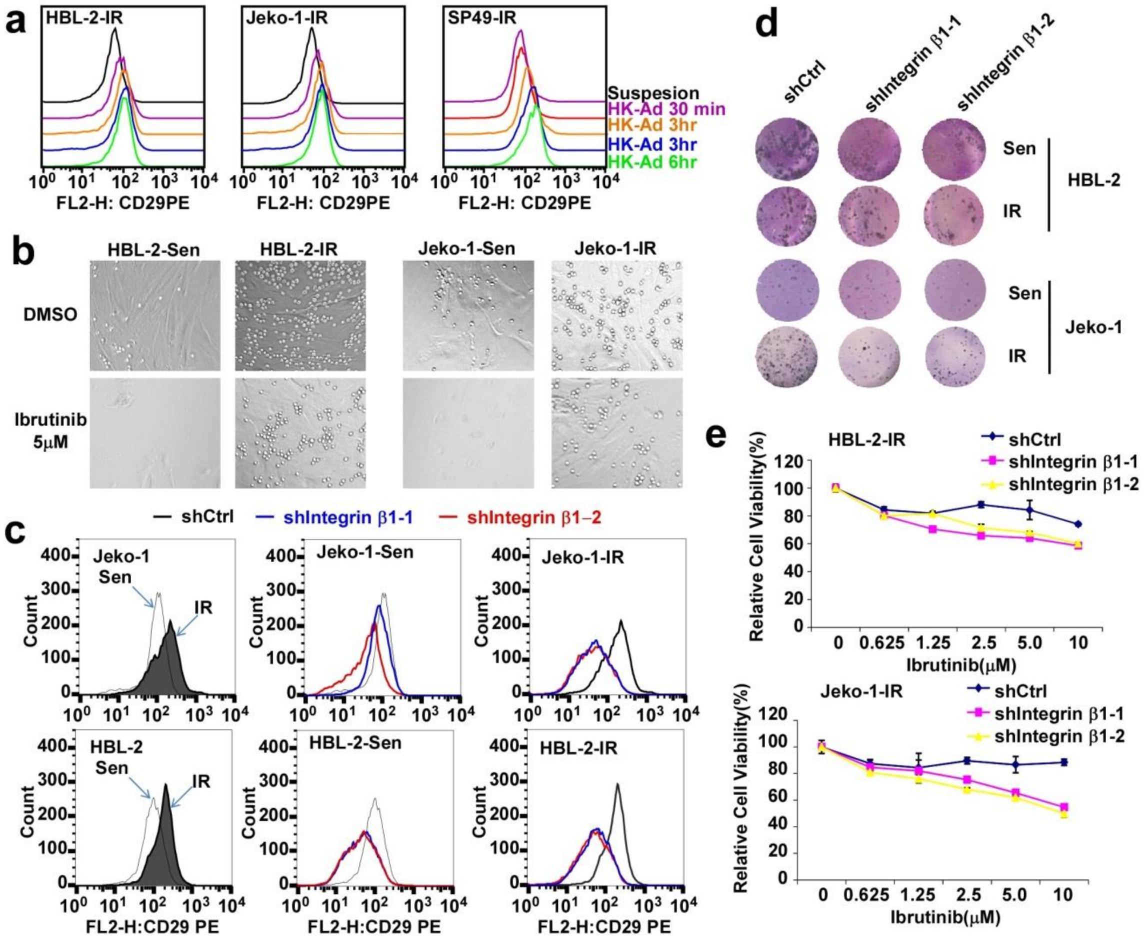

Supplementary Figure 3. Integrin β1 plays important role in cell adhesion, colony formation and cell growth in ibrutinib resistant MCL cells. (a). Co-culture with stromal cells HK up-regulated integrin β1 expression in IR HBL-2-IR, Jeko-1-IR and SP49-IR cells detected by Flow cytometry. **(b).** Cell adhesion assay showing increased and sustained cell adhesion to HK cells in the absence and presence of ibrutinib treatment (12 hours) in IR MCL cells by microscopic examination. **(c).** Flow cytometry confirmed decreased integrin β1 (CD29) expression in stable integrin β1 knockdown cells labeled as shIntegrin β1-1/2. **(d-e).** Stable integrin β1 knockdown significantly decreased colony formation (d) and cell growth/viability in parental (Sen) and IR MCL cells (e). Results in a-d are representatives or mean ± SD from at least three biological replicates.

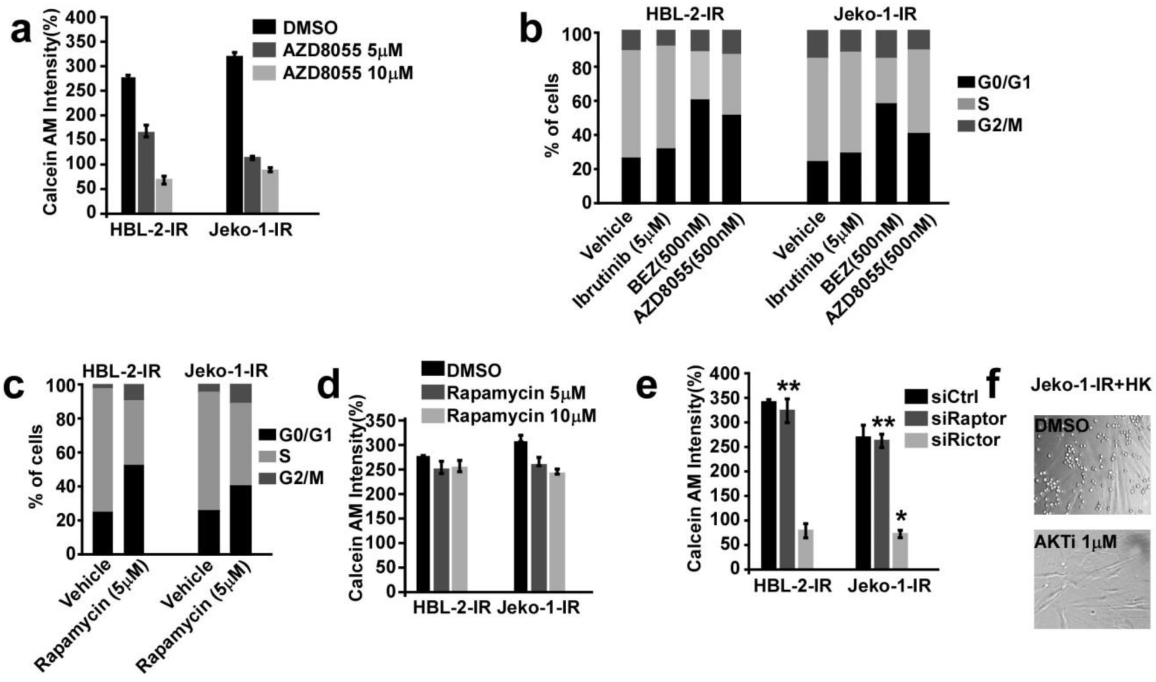

Supplementary Figure 4. Functional dissection of mTORC1 and mTORC2 in MCL cell cycle and adhesion to stroma cells in ibrutinib resistant MCL cells. (a). AZD8055 decreased MCL adhesion to HK cells in IR MCL cells. **(b).** BEZ235 and AZD8055 but not ibrutinib significantly induced cell cycle G1 arrest in IR MCL cells measured by flowcytometry. **(c-d).** Rapamycin induced cell cycle G1 arrest in ibrutinib resistant MCL cells but had no effect on cell adhesion to HK cells. **(e).** Rictor (mTORC2) knockdown by siRNA transfection decreased cell adhesion to HK cells, in contrast, blocking mTOR1 by Raptor knockdown showed no effect on cell adhesion in IR MCL cells**.** * $P<0.05$, ** $P>0.05$. (**f**). AKT inhibitor A674563 significantly attenuated MCL cell adhesion to HK cells in IR MCL cells. a-f, results are representatives of at least three independent experiments or mean ± SD from at least three biological replicates.

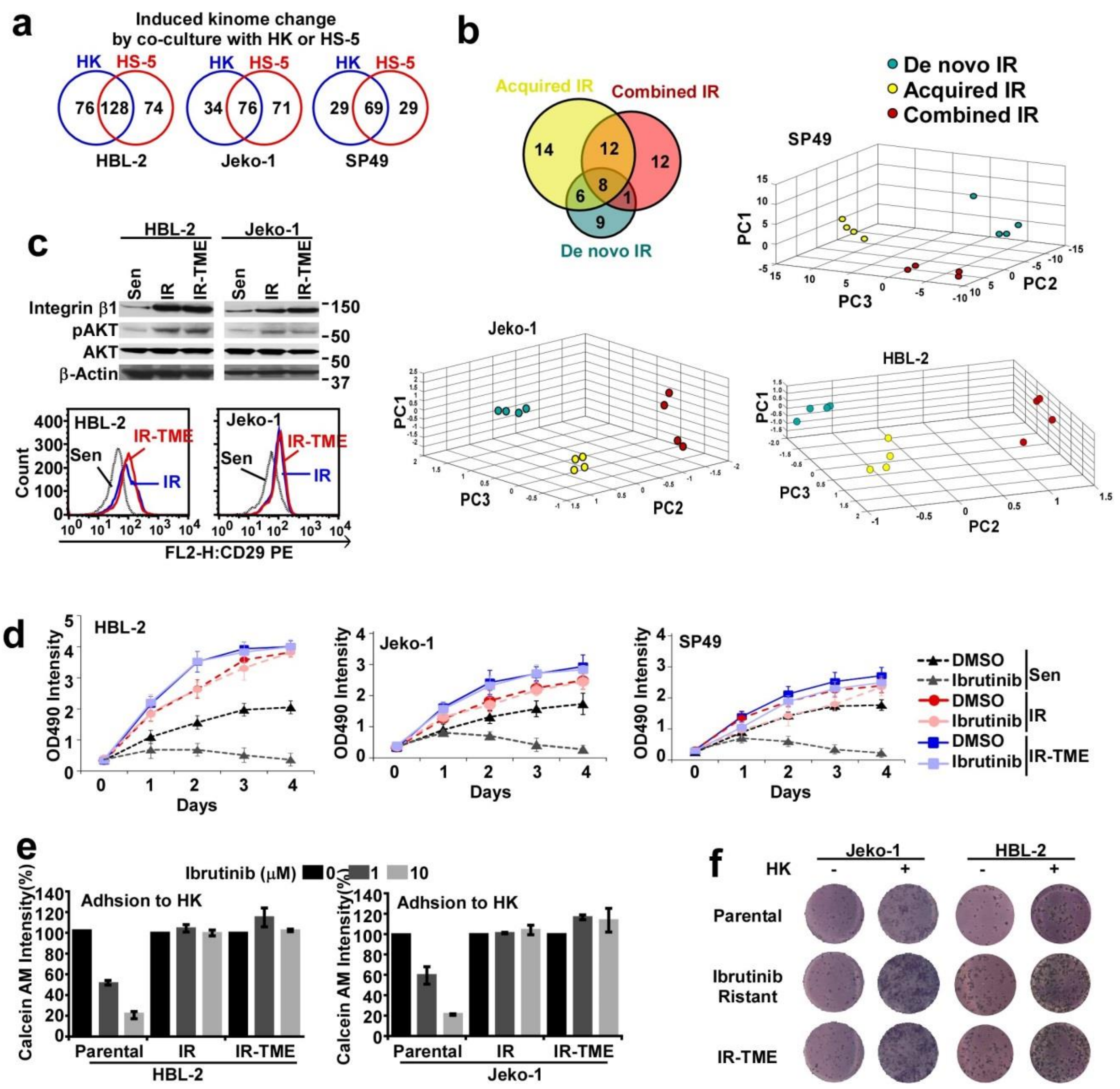

Supplementary Figure 5. Functional coordination between TME-mediated (de novo) and acquired drug resistance provides the unification of TME-mediated and acquired resistance. (a). Comparison of HK-induced kinome with bone marrow stroma HS-5-induced kinome changes showing significant overlaps between these two conditions in all three lines. **(b)**. Venn diagram and principal component analysis (PCA) showing the increased ATP binding kinase and kinomes that were exclusive or common in parental cells (as control), IR cells under chronic ibrutinib exposure while selected in suspension (IR, acquired IR) or adherent to TME stroma (IR-TME, combined IR). **(c).** Increased AKT phosphorylation and β1 expression in parental cells (sen), IR cells under chronic ibrutinib exposure while selected in suspension (IR, acquired IR) or adherent to TME stroma (IR-TME, combined IR). **(d).** Enhanced and comparable cell viability of IR and IR-TME with or without ibrutinib treatment (5 μM for Jeko-1 and HBL-2, 0.2μM for SP49) compared to parental (Sen) cells measured by CCK8. **(e).** Enhanced cell adhesion to HK cells of IR and IR-TME with or without ibrutinib treatment (5 μM) compared to parental cells. **(f)**. Colony formation assay showing more and comparable colony formations in acquired IR cells and IR-TME cells compared to parental cell with or without HK cell co-culture. Results from a to f are representatives of at least three independent experiments or are shown as mean ± SD from at least three biological replicates.

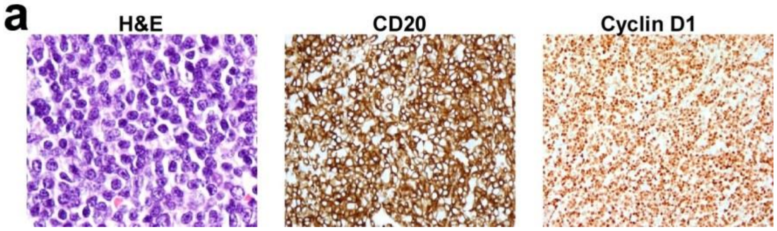

Supplementary Figure 6. (a) Histology and immunohistochemical stains for CD20 and cyclin D1 confirmed recurrent MCL.

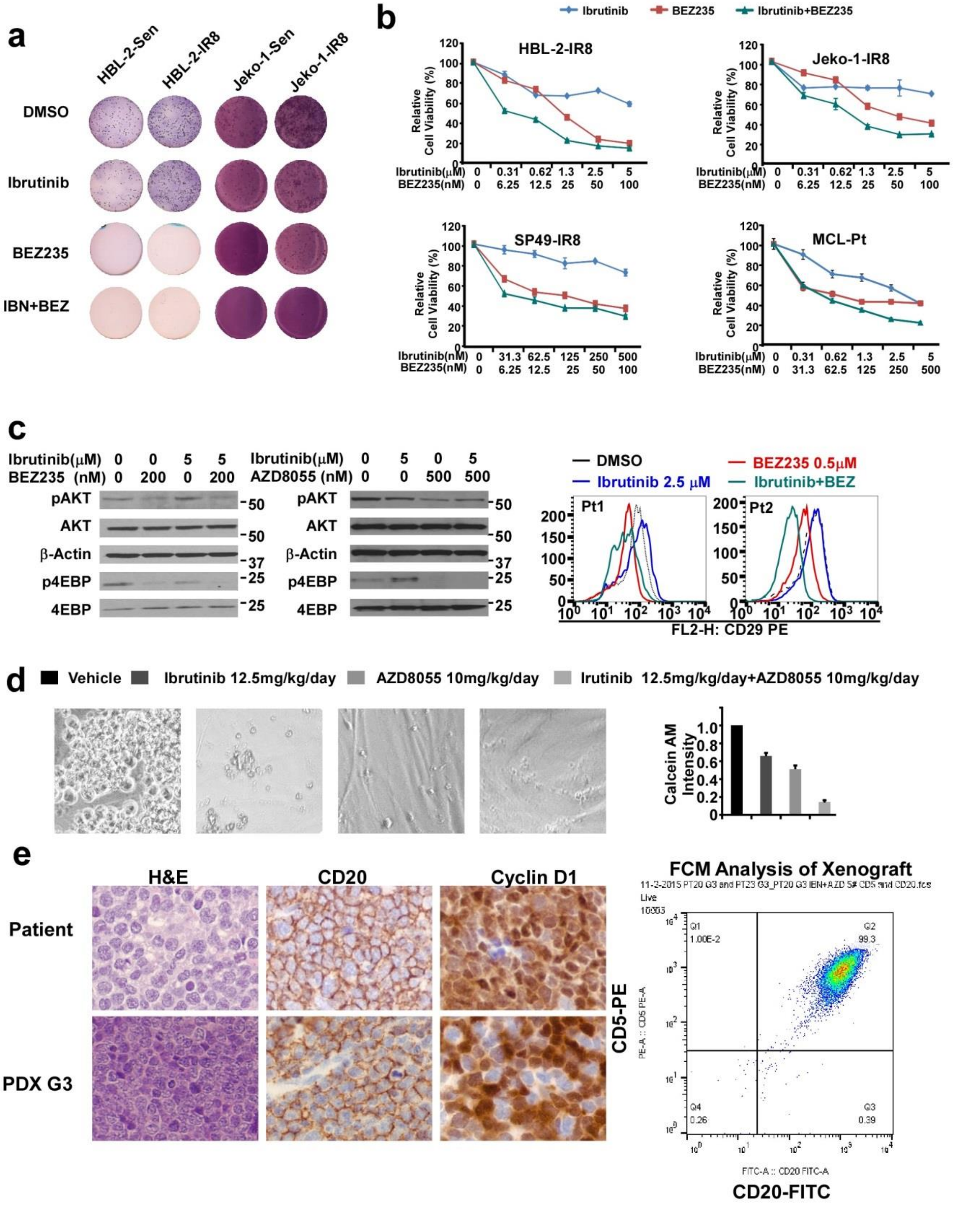

Supplementary Figure 7. Inhibition of PI3K-AKT-mTOR pathway overcomes ibrutinib resistance. (a). BEZ235 in combination with ibrutinib (IBN) more substantially inhibited colony formation in parental (Sen) and IR MCL cells. **(b).** BEZ235 in combination with ibrutinib more substantially inhibited cell viability in IR cell lines and primary IR MCL patient cells measured by CCK8 assay. **(c).** BEZ235 or ADZ8055 more substantially decreased integrin β1 expression and AKT phosphorylation in two primary IR MCL patient samples detected by western blot and flow

cytometry. **(d).** Cell adhesion assay was conducted on the xenograft tumor cells from NOD/SCID mice cohorts as described in Fig. 7d. (e). Histology and immunohistochemical stains for CD20 and cyclin D1 and flow cytometry analysis showing co-expression of CD5 and CD20 on original primary human tumor and PDX tumor confirmed MCLs. a, c and d, results are representative of at least three independent experiments; Results of B are shown as mean $\pm$ SD from at least three biological replicates except for MCL-Pt sample.

HBL-2 co-culture

Jeko-1 co-culture

Full gel scan for Figure 1 a

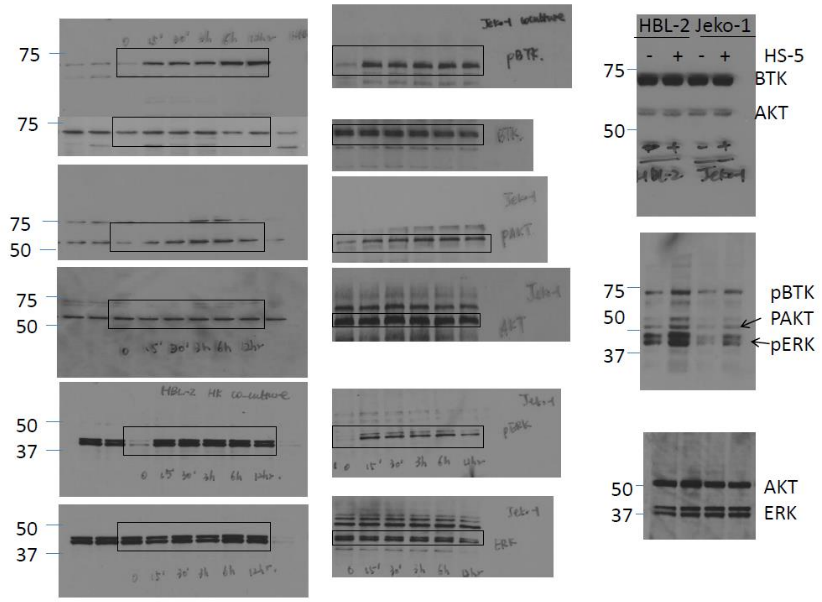

Full gel scan for Figure 1 b

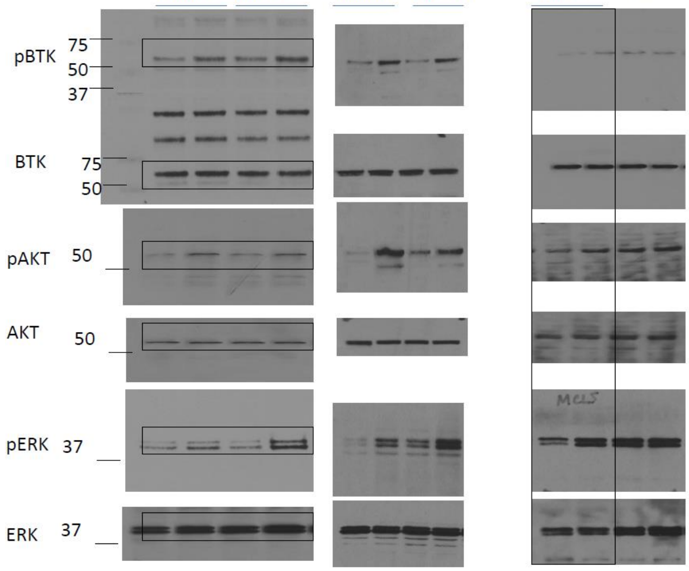

Full gel scan for Figure 1c

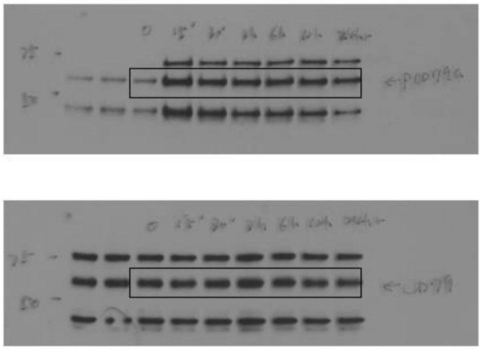

Full gel scan for Figure-1d (SP-49 + HK)

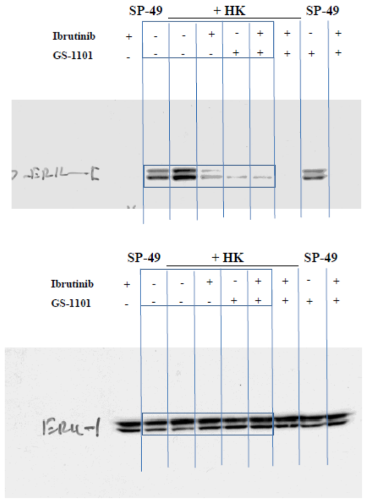

Full gel scan for Figure-1d (Mino + HK)

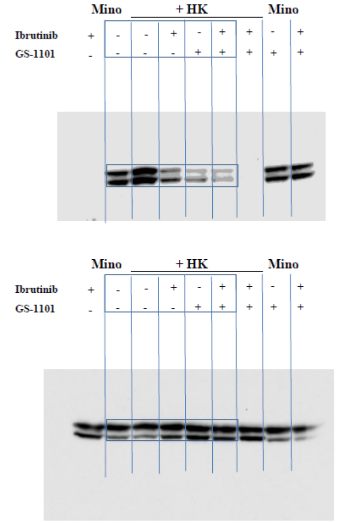

Full gel scan for Figure-1d (SP-49 + HK)

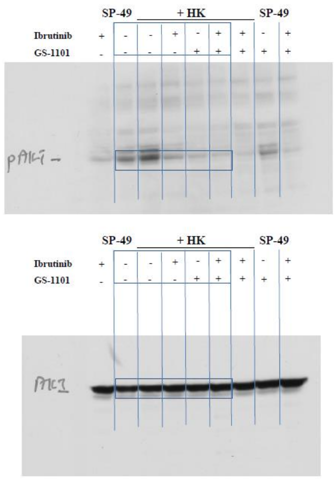

Figure-1d (Mino + HK)

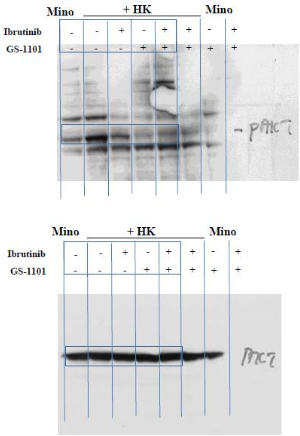

Full gel scan for Figure-1g HBL-2 + HK

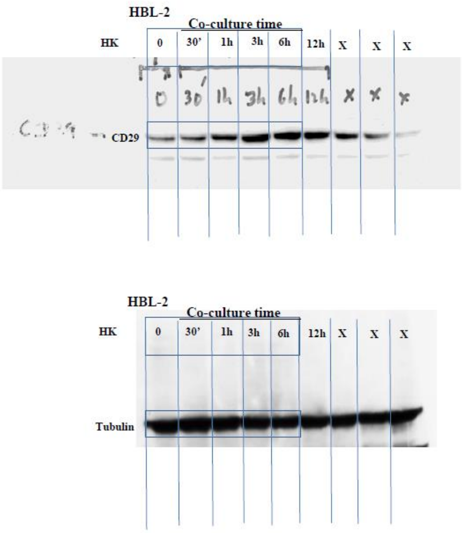

Full gel scan for Figure-1g Jeko-1 + HK

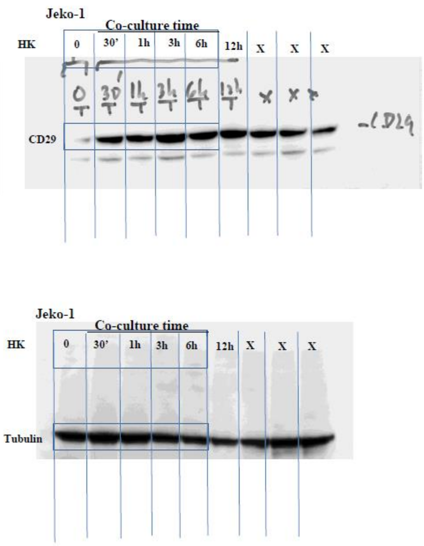

Full gel scan for Figure 1 h

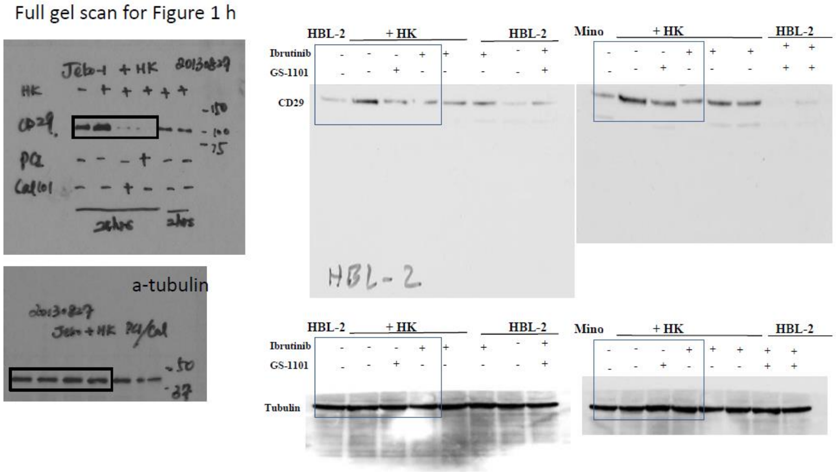

Full gel scan for Figure 2f left panel

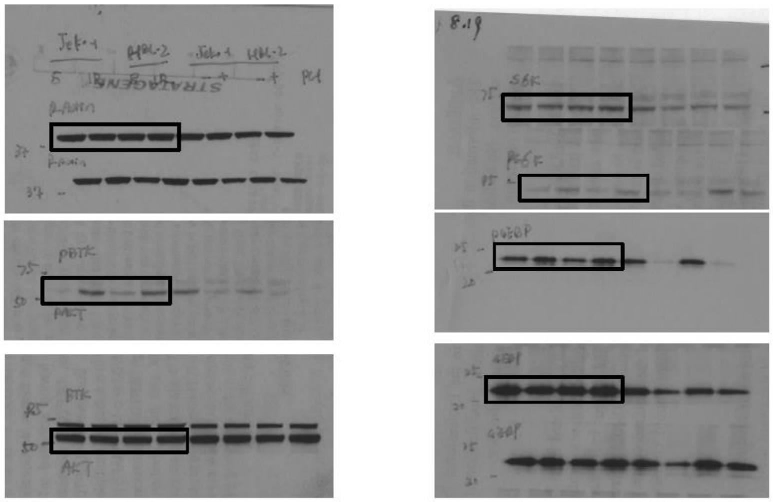

Full gel scan for Figure 2f right panel

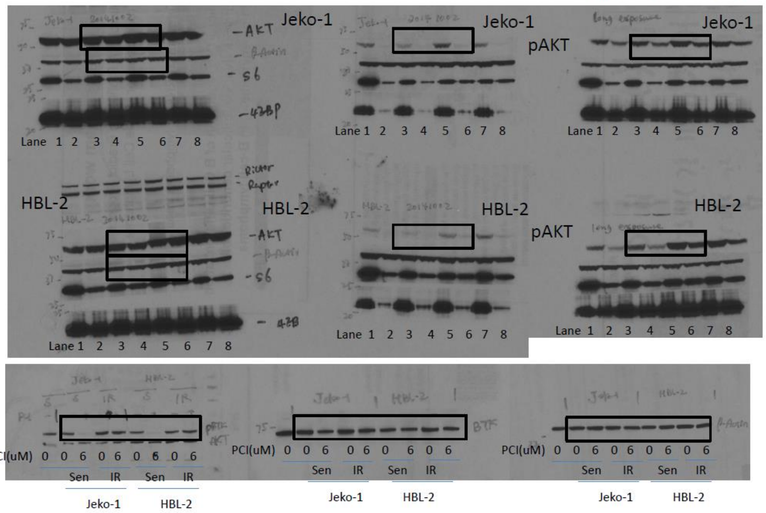

Full gel scan for Figure 3a

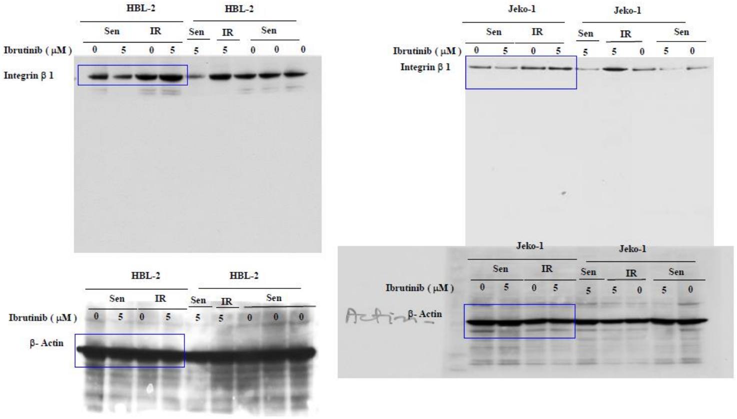

Full gel scan for Fgirue 3 f

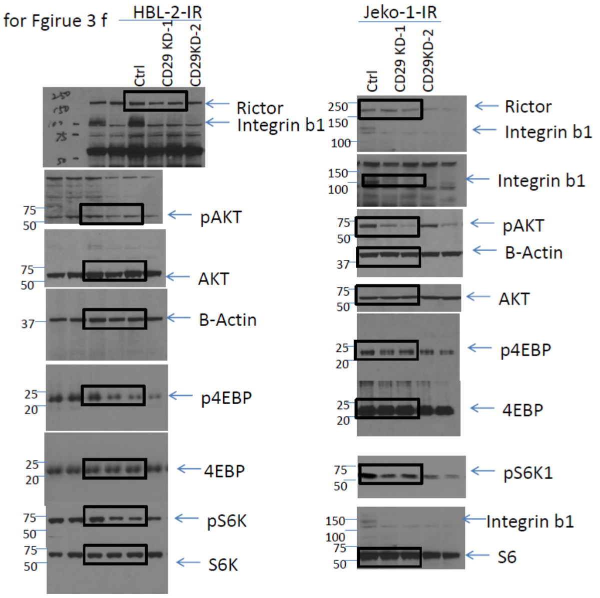

Full gel scan for Figure 3 g

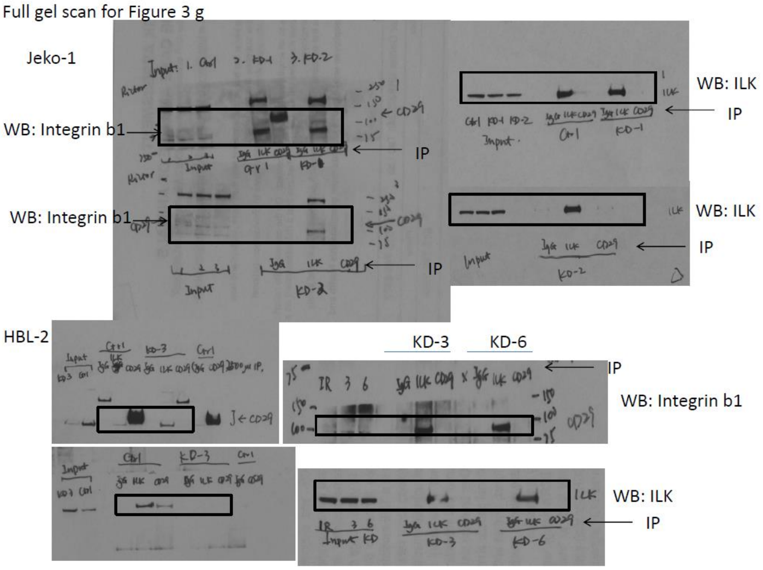

Full gel scan for Figure 3 h

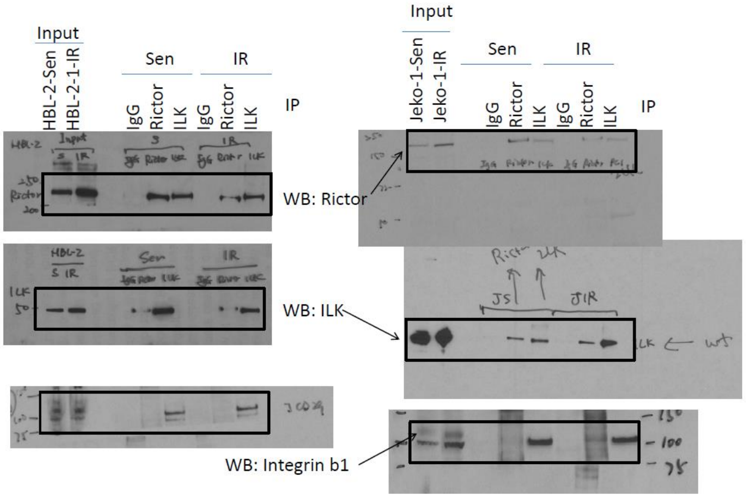

Full gel scan for Figure 3i

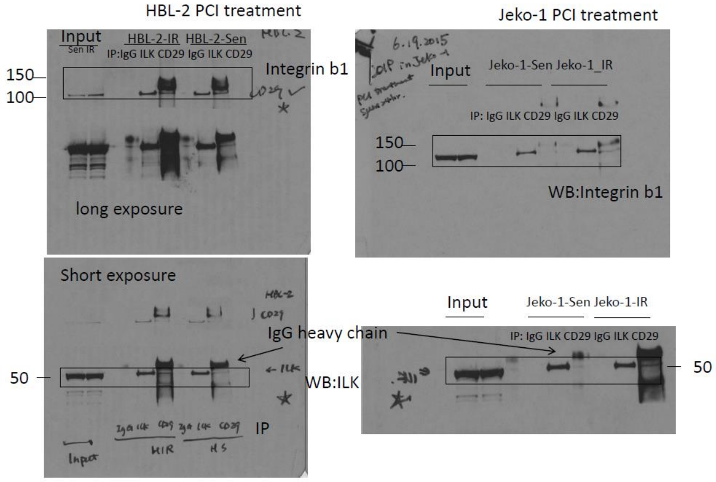

Full gel scan for Figure 4 a Ibrutinib and AZD8055 combination

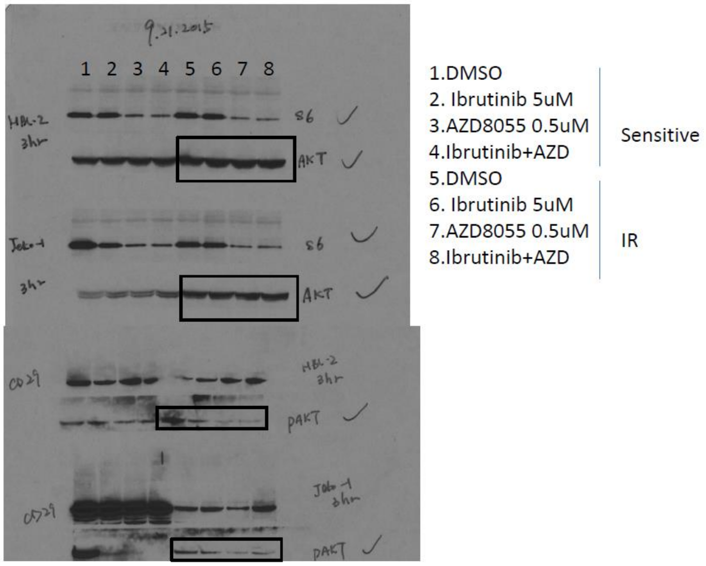

Full gel scan for Figure 4 a Ibrutinib and AZD8055 combination

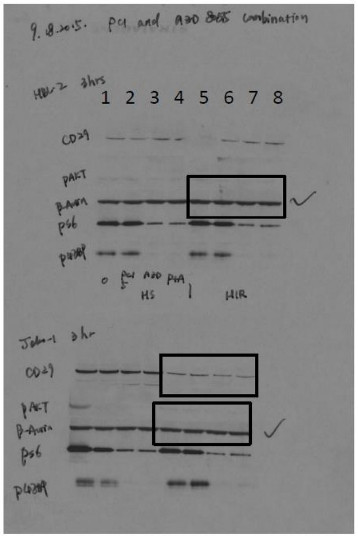

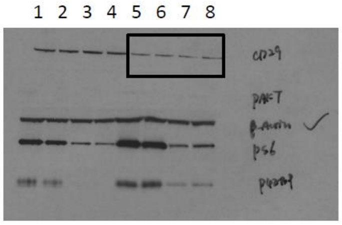

1.DMSO
2. Ibrutinib 5uM
3.AZD8055 0.5uM
4.Ibrutinib+AZD
Sensitive
5.DMSO
6. Ibrutinib 5uM
7.AZD8055 0.5uM
8.Ibrutinib+AZD
IR

Full gel scan for Figure 4a

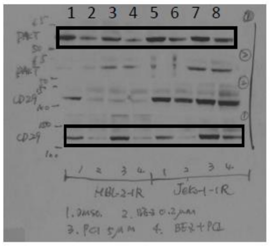

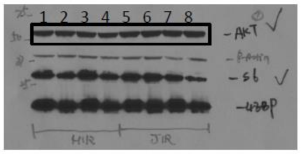

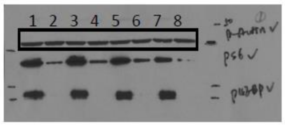

1.DMSO
2.BEZ235 0.2uM
3.Ibrutinib 5uM
4.Ibrutinib+BEZ
HBL-2-IR
5.DMSO
6. BEZ235 0.2uM
7.Ibrutinib 5uM
8.Ibrutinib+BEZAZD
Jeko-1-IR

Full gel scan for Figure 4d

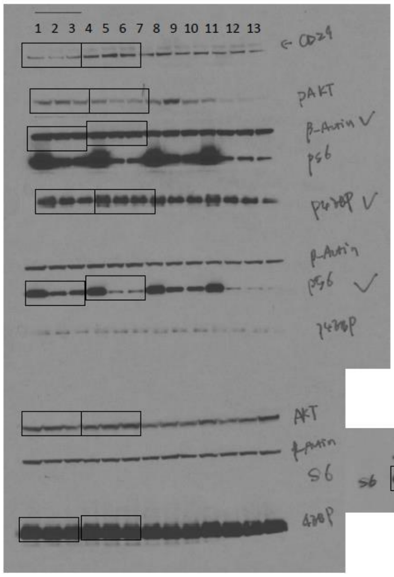

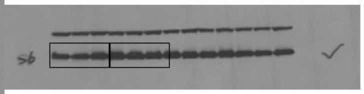

Rapamycin C

1. DMSO
2. 2.5uM
3. 5uM

HBL-2-IR

4. DMSO
5. 2.5uM
6. 5uM

Jeko-1-IR

7. DMSO
8. 2.5uM
9. 5uM

HBL-2-Sen

10. DMSO
11. 2.5uM
12. 5uM
13. 5uM

Jeko-1-Sen

Full gel scan for Figure 4e HBL-2-IR

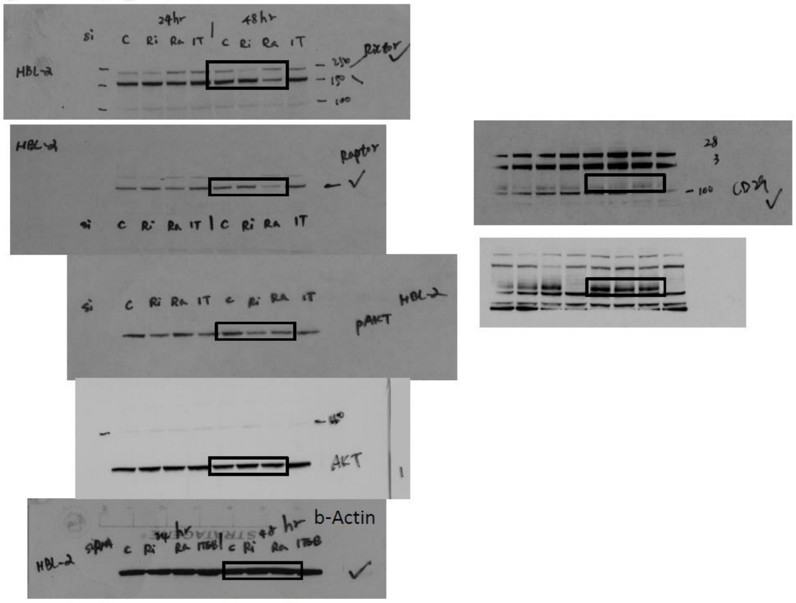

Full gel scan for Figure 4e Jeko--1IR

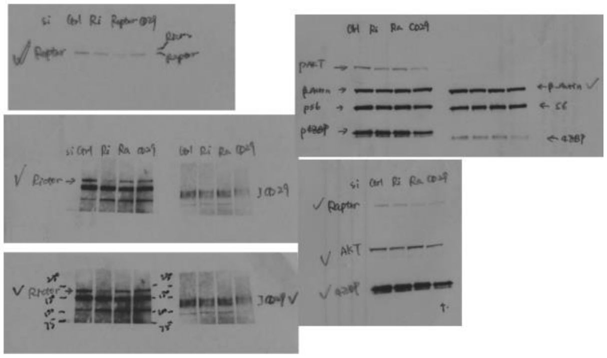

Full gel scan for Figure 6 b

Xenograft 1 2 3 4 5 6 7 8

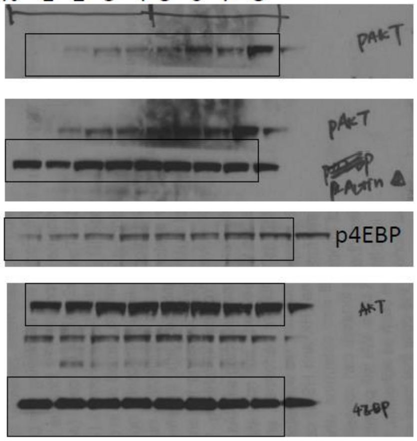

Full gel scan for Figure 7e

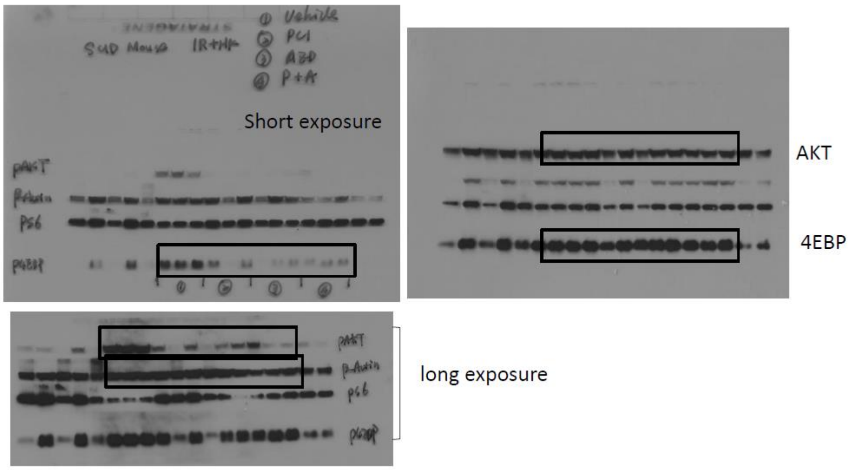

Full gel scan for Figure S1a

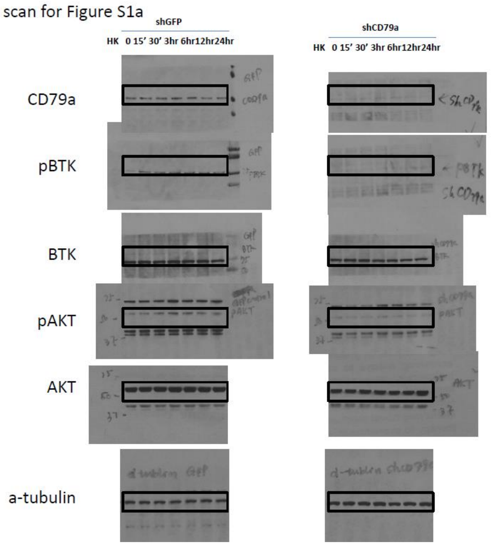

Full gel scan for Supplementary Figure1b

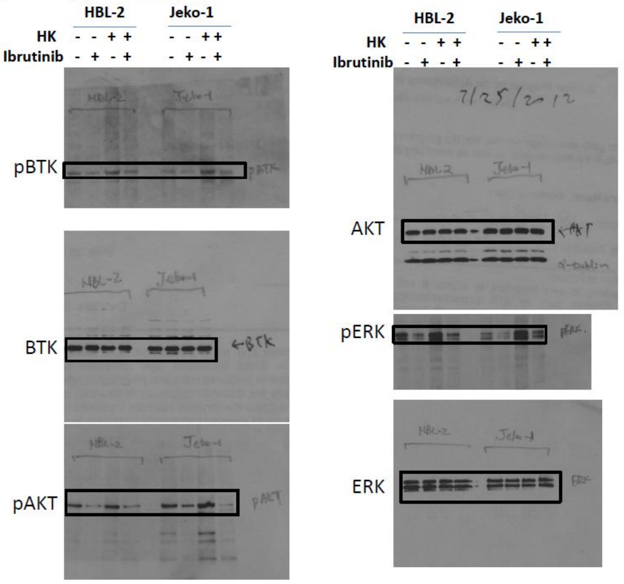

Full gel scan for supplementary Figure -1f
Induction of BTK-AKT-Erk as well as CD79A in Jeko-1 and HBL-2 by FN-Adhesion

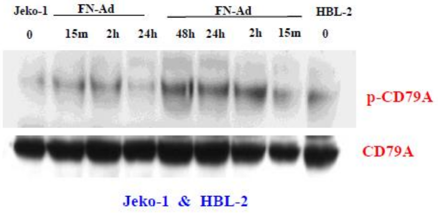

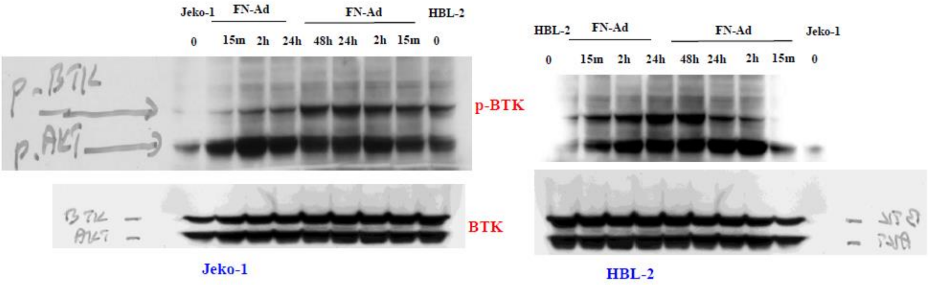

Full gel scan for Supplementary Figure-1f
Induction of BTK-AKT-Erk as well as CD79A in Jeko-1 and HBL-2 by FN-Adhesion

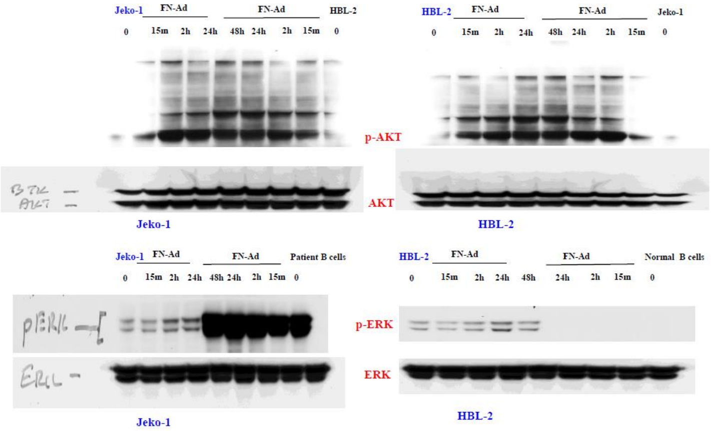

Full gel scan for Supplementary Figure-1f
Induction of BTK-AKT-Erk as well as CD79A in Jeko-1, HBL-2 and SP-49 cells by BAFF.

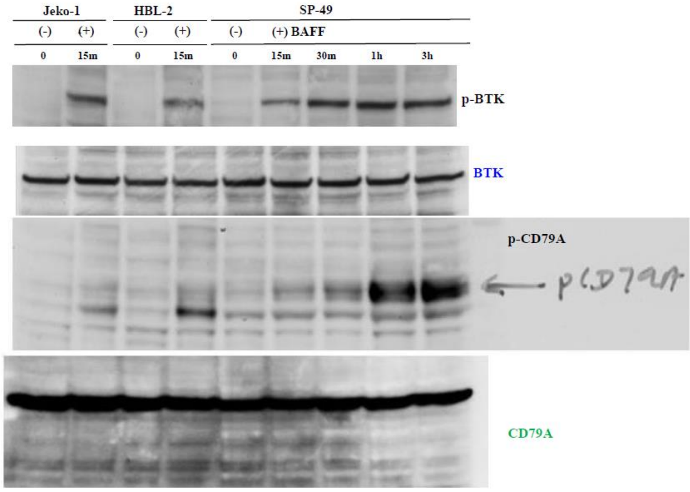

Full gel scan for Supplementary Figure-1f

Induction of BTK-AKT-Erk as well as CD79A in Jeko-1, HBL-2 and SP-49 cells by BAFF.

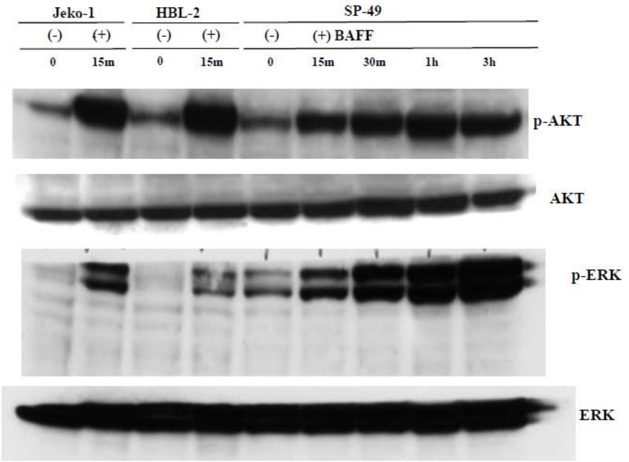

Full gel scan for Supplementary Figure 1g

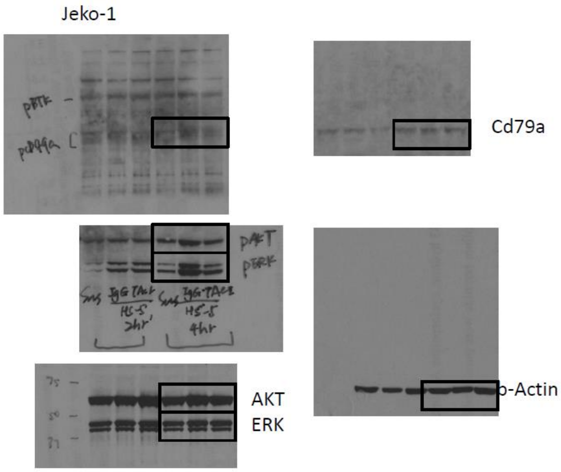

Full gel scan for Supplementary Figure-1g

TACI-Fc blocks Induction of BTK-AKT-Erk as well as CD79A in HBL-2 cells by HK-Ad

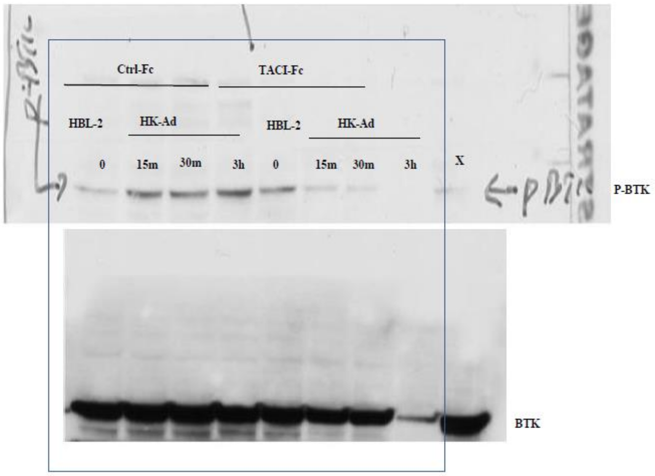

Full gel scan for Supplementary Figure 1g

TACI-Fc blocks Induction of BTK-AKT-Erk as well as CD79A in HBL-2 cells by HK-Ad

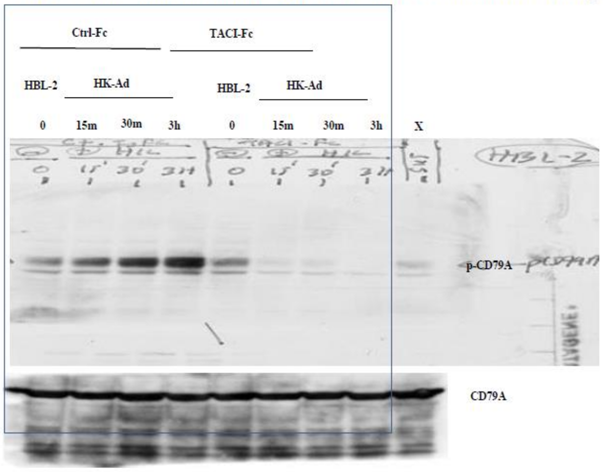

Full gel scan for Supplementary Figure1g

TACI-Fc blocks Induction of BTK-AKT-Erk as well as CD79A in HBL-2 cells by HK-Ad

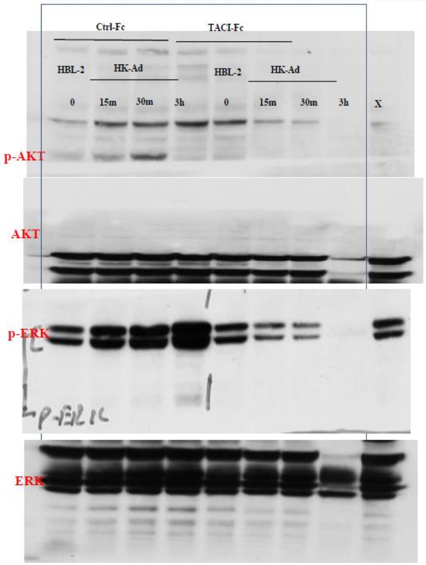

Full gel scan for Supplementary Figure 1h

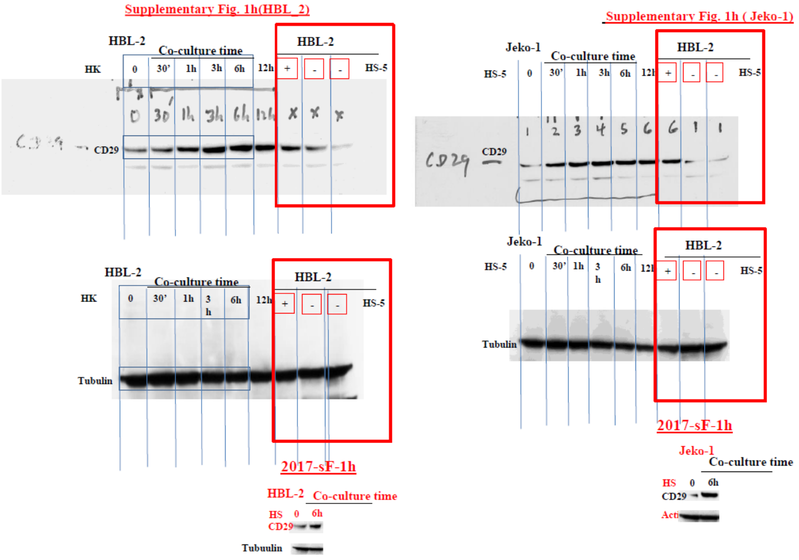

Full gel scan for supplementary Figure 1i

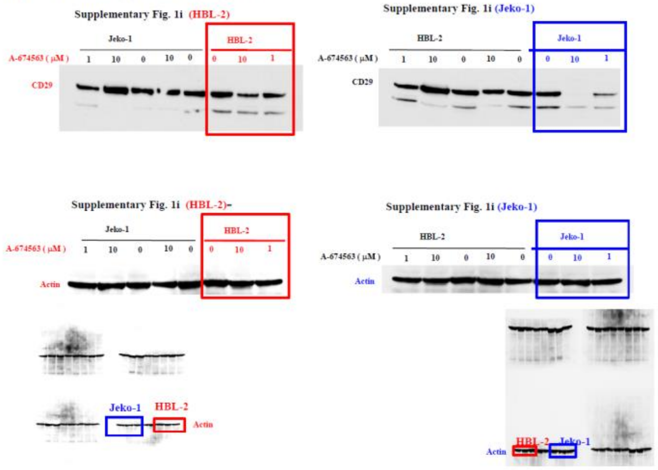

Full gel scan for Supplementary Figure 1j

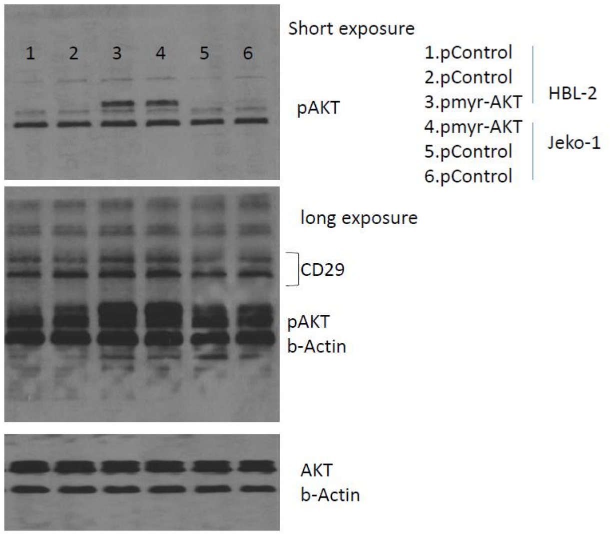

Full gel scan for Supplementary Figure S5c

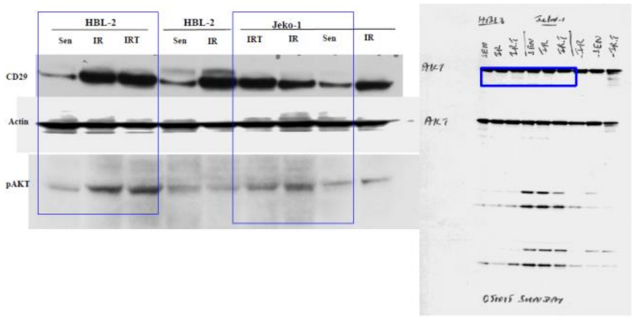

Full gel scan for supplementary Figure 7c

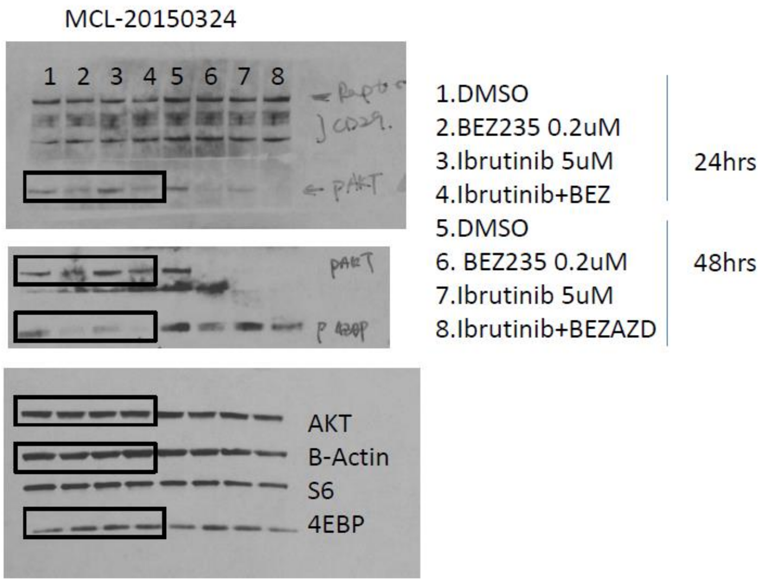

Full gel scan for Supplementary Figure 7c

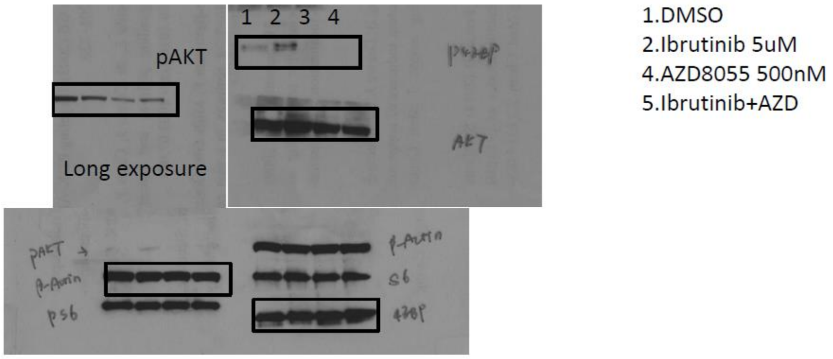

Supplementary Figure 8. Full gel scans of the western blots in figures and supplementary figures.

Supplementary Methods

Cell lines, cell co-cultures, and patient samples.

The human lymphoma cell lines Jeko-1, HBL-2, Mino and stromal cell lines HK, HS-5 were provided and maintained as previously described [2]. SP49 was provided by Dr M. Wasik, University of Pennsylvania. The co-culture experiments were also performed as previously described [2]. The final concentration of BAFF is 400ng/ml and TACI-Fc/IgG-Fc is 20ng/ml in culture medium for BCR activation or inhibition experiments.

For co-culture experiments, either lymphoma cell lines or patient lymphoma cells (1.5 times 10^6 cells per ml) were added and adhered to pre-established monolayers of HS-5 or HK cells or were kept in suspension for 12–24 h. Then, lymphoma cells were carefully removed by gentle shaking, with the monolayer of ~80% confluent HS-5 and HK cells kept intact. The purity of the lymphoma cell population was confirmed to be greater than 90% when analyzed by flow cytometry (CD19 vs CD44), and cells were used for experiments as described. For transwell experiments, lymphoma cells were separated from HS-5 by transwell inserts. The patient lymphoma cells designated as MCL were obtained from diagnostic fresh biopsy-derived lymphoma tissues or peripheral blood. This study was conducted in accordance with the protocol approved by the Institutional Review Board. Informed consent was obtained from all patients. After Ficoll-Hypaque purification, if tumor cells accounts for less than 80% of total cells, the CD19-positive cells were positively isolated by using CD19 microbeads with the autoMACS magnetic cell sorter according to the manufacturer's instructions (Miltenyi Biotec, Inc., Auburn, CA, USA).

Selection of drug-resistant cell lines

To establish the ibrutinib resistant cell lines, MCL cells (HBL-2, Jeko-1, SP49) were grown in RPMI-1640 medium with 10% FBS and treated with vehicle control or escalating dose of ibrutinib for approximately 6 months. Before exposing MCL cells to ibrutinib, cells were adhered to either stroma (HK cells) or maintained in suspension. Trypan blue staining and CCK8 viability assay in both suspension and stroma co-cultured cells were then used to determine viability every 2-3 weeks, and ibrutinib concentrations were increased if the viability of cells in both suspension and stroma-co-culture was >65%. If cell viability was <65% in either the suspension or stroma-adhered populations, both populations were allowed to expand in suspension in drug-free media and then re-exposed to the same concentration of ibrutinib or re-exposed to stromal cells after 1:2 split. After ~100 days, drug-resistant variants emerged, and IC50s were determined every two weeks. Following another 80 days of selection, stable variants emerged referred to as either MCL-IR (selected in suspension culture) or MCL-IR-TME (selected while adherent to stroma). Drug-resistant variants were maintained in drug free media for 1 week before utilized in experiments. MCL cells exposed to DMSO were maintained in parallel and used for comparison with their respective drug-resistant cell lines. We used at least more than 4X IC50 (ranging from 4 to 200 fold) as threshold to define the resistance given that the dose resistance curves are flat in some ibrutinib resistant cells.

Plasmids

Human integrin b1 shRNA lentiviral particle (sc-35674-V), control shRNA lentiviral particles (sc-108080), Raptor shRNA plasmid (sc-44069-SH) and Rictor shRNA plasmid (sc-61478-SH) CD79A shRNA plasmid (sc-35026-SH) were purchased from Santa Cruz Biotechonology.

Short-hairpin RNA-mediated gene knockdown

Transient transfection of shRNA plasmid was performed as described previously [3].HBL-2 cells were transfected using Nucleofector KIT V (program O-006). Jeko-1 cells were transfected using Nucleofector KIT V (program X-001).

To establish stable integrin β1 knockdown MCL cell lines, HBL-2, Jeko-1 cells were transduced with lentivirus particles according to the manufacture's protocol (Santa Cruz technology); Forty-eight hours after the transduction, puromycin (Sigma Aldrich) was added to the growth medium starting from 0.5mg/ml until the final concentration of 2mg/ml. After 2 weeks, these transduced cells were analyzed by western blot or flow cytometry to confirm integrin β1 knockdown.

Antibodies

Antibodies used for western blot: pAKT(Ser476), #9271(1:500 dilution); AKT, #9272(1:1000 dilution); BTK(Tyr223), #5082(1:500 dilution); BTK(D3H5),#8547(1:1000 dilution); p44/42 MAPK (Erk1/2) (Thr202/Tyr204) (1:2000 dilution), #4370; p44/42MAPK(Erk1/2)(137F5), #1695(1:2000 dilution); α-Tubulin, #2144(1:1000 dilution), p4E-BP1 (Thr37/46) (236B4), #2855(1:2000 dilution); 4E-BP1 (53H11) ,#9644(1:2000 dilution); S6K(49D7), #2708(1:1000 dilution); pS6K1(Thr421/Ser424), #9204(1:1000 dilution) were from Cell Signaling Technology; β-actin antibody (A1978; Sigma)(1:3000 dilution).

Antibodies for Co-immunoprecipitation (Co-IP) assay are integrin b1 (ab52971, Abcam); RICTOR (ab56578); ILK (65.1) AC (sc-20019 AC, Santa Cruz); normal mouse IgG (sc-2025, Santa Cruz).

Ibrutinib (S2680), idealisib (S2226), A-674563 (S2670), carfizomib (S2853), SCH-772984 (S7101), NVP-BEZ-235 (S1009), AZD8055 (S1555), mitoxantrone (S1889) and doxorubicin (S1208) were purchased from Selleckchem. At the indicated times, cells

were collected and washed, and Western blot or Co-IP as well as apoptosis analyses were then performed as previously described [3].

Immunofluorescent staining

MCL cells were plated on glass coverslips in six-well plates and incubated overnight prior to treatment. Cells were then fixed using 4% paraformaldehyde solution (Electron Microscopy Sciences, Hatfield, PA, USA) and permeabilized with 0.2% Triton X-100 prior to being blocked in 1% bovine serum albumin (BSA)/phosphate-buffered saline (PBS). Coverslips were incubated with primary antibodies overnight at 4 °C. Coverslips were then washed in PBS, incubated with secondary antibodies for 1 h at room temperature, and washed again in PBS and sterile water. Coverslips were mounted with ProLong Gold antifade reagent with 4',6-diamidino-2-phenylindole (Life Technologies) and imaged using confocal microscopy.

Cell function assays

5×10^3 cells were dispensed per well in 96-well plates and different concentration of drugs was added into the wells. Then the cell numbers at indicated time point were detected using CCK-8 Kit according to the manufacture's protocol (CK04-13, Dojindo Technologies, Inc.).

Cell adhesion assay was performed using Vybrant® Cell Adhesion Assay Kit (V-13181, Thermo fisher Scientific) according to the manufacture's protocol. Briefly, HK cells were plated onto six-well plates, and MCL cells labeled with calcein AM were allowed to adhere to the HK cells. After indicated time period, non-adherent cells were removed, and then calcein AM fluorescence was used to calculate the number of adherent cells. For the colony formation assay, 2×10^3 cells (0.05ml) were added to 0.5 ml MethoCult®

(STEMCELL) per well in 24-well plates. After 8-10 days culture, number of colonies were counted and picture of the colonies were taken daily.

Tumoregenesis Assays

Six- to eight-week-old male NOD/SCID mice were purchased from the Jackson Laboratory. All experimental procedures and protocols had been approved by the Institutional Animal Care and Use Committee of The University of South Florida. 10^6 HBL-2 parental or IR cells were injected into the lower flank of NOD/SCID mice with or without 5×10^5 HK cells in a volume of 0.1 mL. And then mice were randomized into control and treatment groups when tumor volume reached 0.2 cm^3 (4 mice per group). AZD8055 and Ibrutinib were formulated in 30% (w/v) Captisol (pH 3.0). The control group received the vehicle only. AZD8055 was given oral lavage daily at a dose of 20mg/kg. Ibrutinib was given oral lavage daily at a dose of 12.5mg/kg. Tumor size was assessed every the other day using a digital caliper. Tumor volumes were determined by measuring the length (l) and the width (w) of the tumor and calculating the volume ($V = l *w^2/2$).

Patient derived xenograft (PDX) model

Primary MCL cells were isolated from lymph node of patients and injected into human bone chips and performed as previously described [4] . Briefly, six- to eight-week-old male CB-17 SCID mice (Harlan) were housed and monitored in our animal research facility. All experimental procedures and protocols had been approved by the Institutional Animal Care and Use Committee of The University of Texas M. D. Anderson Cancer Center. Procedures for SCID mouse implantation (SCID-hu hosts) with human fetal bone grafts have been previously described [4]. Briefly, Wang et al adopted the SCID-hu mouse model developed for myeloma and injected primary human MCL cells isolated from

peripheral blood, lymph node, spleen or bone marrow directly into the microenvironment of human fetal bone, which had been s.c. implanted into SCID mice. Four to eight weeks after inoculation, a large tumor mass was observed around the implanted human bone, and histological examination and immunohistochemical staining with anti-human CD20 and cyclin D1 antibodies confirmed MCL and revealed that patient's MCL cells were able to not only survive and propagate in the bone marrow microenvironment of the human fetal bone chips, but also similar to the human disease, migrate to lymph nodes, spleen, bone marrow, and gastrointestinal tract of host mice. The engraftment of primary MCL cells was shown to produce measurable levels of human β2-microglobulin (β2M) in mouse serum. This model and β2M as a biomarker was further applied to test the in vivo therapeutic efficacy of antitumor compounds for MCL therapy[5]. Here, approximately 4 to 6 wk after implantation, one injection of 0.5 to 5 × 10^6 purified patient MCL cells isolated from abdominal mass of a 74-year-old lady who progressed after ibrutinib treatment, was administered directly into human fetal bone implants within SCID-hu hosts or i.v. into tail vein after mice were anesthetized with ketamine (75 mg/kg) and xylazine (12.5 mg/kg; Lloyd) [4]. PDXs were established in SCID-hu mice and propagated in NSG mice. The NSG mice bearing the PDXs were randomly divided into 6 groups (4/group) and were treated with vehicle, ibrutinib 25 mg/kg oral gavage daily alone, AZD8055 5 mg/kg oral gavage daily alone, NEP-BEZ235 10 mg/kg oral gavage daily alone, ibrutinib +AZD8055 (ibrutinib 25 mg/kg oral gavage daily with AZD8055 5 mg/kg oral gavage daily) and ibrutinib+BEZ (ibrutinib 25 mg/kg oral gavage daily with NEP-BEZ235 10 mg/kg oral gavage daily). Tumor volumes were examined before treatment started (day 0) and at days 12 and 19. The critical MCL phenotypes of the PDX tumor shown such as CD20/CD5 and Cyclin D1 were determined by histology, flow cytometry and immunostains and shown in Fig S7E.

Activity-Based Protein Profiling (ABPP)

Cell pellets were sonicated briefly in IP/Lysis buffer containing 25 mM Tris-HCl pH 7.4, 150 mM NaCl, 1 mM EDTA, 1% NP-40 and 5% glycerol and centrifuged to clear the lysate. The supernatant was passed through a Zeba spin desalting column (Catalog # 89891, Thermo), which had been conditioned 3 times with reaction buffer containing 20 mM HEPES, 150 mM NaCl, 0.1% Triton X-100 at pH 7.4. An aliquot (1 mg) of total protein was labeled with 10 µM desthiobiotin-ATP reagent (catalog # 88310, Thermo) at room temperature for 10 minutes. After reduction with 500 mM DTT at 65 ºC for 30 minutes and alkylation with 1 M iodoacetamide in darkness for 30 minutes, the labeled proteins were passed through the Zeba spin desalting column, which has been conditioned 3 times with digestion buffer containing 2 M urea, 20 mM Tris, pH 8.0 and trypsin in solution digestion was carried out at 37 ºC for two hours with enzyme to protein ratio of 1:50. The labeled peptides were captured with high capacity streptavidin agarose resin at room temperature for 1 hour. After 3 washes with IP/Lysis buffer, 4 washes with PBS and 4 washes with H_2O, the peptides were eluted with 50% acetonitrile with 0.1% trifluoroacetic acid. After Speedvac concentration, the peptides were re-dissolved in aqueous 2% acetonitrile with 0.1% trifluoroacetic acid. For drug treatment experiment, the cells were treated for 6 h prior to collection with 2 µM ibrutinib to inhibit BTK.

LC-MS/MS

A nano flow liquid chromatograph (RSLC, Dionex, Sunnyvale, CA) interfaced with an electrospray ion trap mass spectrometer (LTQ-Orbitrap or Q-Exactive Plus, Thermo, San Jose, CA) was used for tandem mass spectrometry peptide sequencing experiments and relative quantification of ATP probe uptake. The sample was first loaded onto a pre-column (2 cm x 100 µm ID packed with PepMap C18 reversed-phase resin, 5µm, 100Å)

and washed for 8 minutes with aqueous 2% acetonitrile containing 0.04% trifluoroacetic acid. The trapped peptides were then eluted onto the analytical column, (C18, 75 µm ID x 50 cm packed with PepMap C18 reversed-phase resin, 2 µm, 100Å, Dionex, Sunnyvale, CA). The gradient was programmed as: 95% solvent A (2% acetonitrile + 0.1% formic acid) for 8 minutes, solvent B (90% acetonitrile + 0.1% formic acid) from 5% to 50% in 90 minutes, increasing from 50% to 90% B in 7 minutes, then held at 90% for 5 minutes. Re-equilibration was achieved by decreasing solvent B from 90% to 5% in 1 minute and holding at 5% B for 10 minutes. The flow rate for the analytical column was 300 nl/min. For the LTQ-Orbitrap or Q-Exactive Plus, either 5 or 16 tandem mass spectra were collected in a data-dependent manner following each survey scan, respectively. In both instruments, the MS scans were acquired in the Orbitrap with AGC target set to 1,000,000 to obtain accurate peptide mass measurements, and the MS/MS scans were acquired using with AGC target set to 30,000 (LTQ-Orbitrap, linear ion trap) and 500,000 (Q-Exactive Plus), respectively. Previously sampled peptide peaks are excluded from MS/MS for 60 seconds.

Database Searching and Quantification

MaxQuant [6] was used to identify and quantify peptides. The precursor mass tolerance was set at 10 ppm for Orbitrap data. Dynamic modifications included carbamidomethylation (Cys), oxidation (Met), and Desthiobiotin. Peptide identification FDR was set to 0.05. Swiss-Prot human database was downloaded in July, 2015. The identification of peptides with significant changes was manually verified. Data normalization was performed using iterative rank order normalization (IRON), software implementation of IRON is available at [http://gene.moffitt.org/libaffy/][7].

Go enrichment analysis and KEGG pathway visualization

Fold-change distribution of the ATP binding proteome was performed by GraphPad software. Go enrichment analysis was performed on the ABPP profile using PANTHER classification system [1]. Kyoto Encyclopedia of Genes and Genomes (KEGG) enrichment analysis was performed on the increased protein kinases (comparing ibrutnib resistance and parental cells) from 2 out of 3 cell lines. Gene enrichment analysis of secretome experiment was done by Gene Go (Thomson Reuters Systems Biology Solutions).

Statistics

One-tailed Student's t test for homoscedastic variances was used to evaluate RT-PCR and apoptosis assay data. Statistical significance between the control and treated mice was evaluated using a 1-tailed Student's t test. P values of less than 0.05 were considered significant. Data are shown with the mean ± SD of at least 4 experiments.

Study approval

The human specimen study was approved by the Institutional Review Board at the University of South Florida, and patients provided signed informed consent forms.

Supplementary References

1. Mi, H., Muruganujan, A., Casagrande, J.T. & Thomas, P.D. Large-scale gene function analysis with the PANTHER classification system. *Nat Protoc* **8**, 1551-1566 (2013).
2. Lwin, T.*, et al.* A microenvironment-mediated c-Myc/miR-548m/HDAC6 amplification loop in non-Hodgkin B cell lymphomas. *The Journal of clinical investigation* **123**, 4612-4626 (2013).
3. Zhang, X.*, et al.* Coordinated silencing of MYC-mediated miR-29 by HDAC3 and EZH2 as a therapeutic target of histone modification in aggressive B-Cell lymphomas. *Cancer cell* **22**, 506-523 (2012).
4. Wang, M.*, et al.* A severe combined immunodeficient-hu in vivo mouse model of human primary mantle cell lymphoma. *Clinical cancer research : an official journal of the American Association for Cancer Research* **14**, 2154-2160 (2008).
5. Zhang, L.*, et al.* In vitro and in vivo therapeutic efficacy of carfilzomib in mantle cell lymphoma: targeting the immunoproteasome. *Molecular cancer therapeutics* **12**, 2494-2504 (2013).
6. Cox, J. & Mann, M. MaxQuant enables high peptide identification rates, individualized p.p.b.-range mass accuracies and proteome-wide protein quantification. *Nat Biotechnol* **26**, 1367-1372 (2008).
7. Welsh, E.A., Eschrich, S.A., Berglund, A.E. & Fenstermacher, D.A. Iterative rank-order normalization of gene expression microarray data. *BMC bioinformatics* **14**, 153 (2013).
